# Parents’ experiences of life after medicalised conception: a thematic meta-synthesis of the qualitative literature

**DOI:** 10.1186/s12884-023-05727-x

**Published:** 2023-07-17

**Authors:** Z. Foyston, L. Higgins, D. M. Smith, A. Wittkowski

**Affiliations:** 1 School of Health Sciences, The University of Manchester, Manchester, UK; 2 Greater Manchester Mental Health NHS Foundation Trust, Manchester, UK; 3 Maternal and Fetal Health Research Centre, University of Manchester, Manchester, UK; 4 St. Mary’s Hospital, Manchester University Hospital NHS Foundation Trust, Manchester, UK; 5 Division of Psychology and Mental Health, School of Health Sciences, Faculty of Biology, Medicine and Health, the University of Manchester, Manchester Academic Health Science Centre, 2nd Floor Zochonis Building, Manchester, UK; 6 Manchester Academic Health Science Centre, Manchester, UK

**Keywords:** Pregnancy, Parenting, Antenatal, Postnatal, Assisted reproduction, Systematic review

## Abstract

**Background:**

Medicalised Conception (MAC) assists many couples to achieve pregnancy worldwide. As the impact of MAC has been linked to increased pregnancy-specific anxiety and parenting difficulties, this review aimed to explore parental experiences of pregnancy and early parenting following MAC, identifying parents’ psychological, social and health needs.

**Method:**

Five databases were searched systematically from inception to March 2023. Identified articles were screened for eligibility against the inclusion criteria and the results were analysed using thematic synthesis. The Critical Appraisal Skills checklist was employed to appraise methodological quality.

**Results:**

Twenty qualitative studies, drawing on a total of 19 participant samples, were included in this review, most with samples with history of subfertility. The findings were synthesised into three main themes (consisting of seven subthemes): 1) *The vulnerable parent: fear, doubt, uncertainty,* 2) *the stark realisation of the parental dream,* 3) *psychosocial needs and support.* Parents lacked a sense of safety during pregnancy and reported acting protectively both antenatally and postnatally*.* Furthermore, their identity transition was complex and non-linear, influenced by sociocultural context.

**Conclusions:**

Considerable unmet psychosocial needs were identified including the potential for anxiety in pregnancy, the possibility of feeling excluded and marginalised, and a reluctance to share distress and experiences with healthcare professionals. These findings suggest a need for consistent, holistic care, integrating psychological services.

**Supplementary Information:**

The online version contains supplementary material available at 10.1186/s12884-023-05727-x.

## Introduction

Medicalised conception (MAC) assists individuals in achieving pregnancy. MAC is an umbrella term which encompasses many different types of treatment including In-Vitro Fertilisation (IVF), Intracytoplasmic Sperm Injection (ICSI), Donor Insemination and Gamete Intrafallopian Transfer [1]. Most individuals who seek MAC are heterosexual couples with fertility challenges (96%) [1].

The prevalence of MAC is rising year on year, for example, with IVF birth rates three times higher in the United Kingdom (UK) when compared to 1991 [1]. In 2019, 2.1% of all births in the United States resulted from MAC [2]; similar statistics are seen in many other countries [3, 4]. MAC is often not a singular event but a repeated cumulative process [5]. The physical and emotional demands of MAC are well documented in the literature, impacting subsequent antenatal and postnatal experiences when conception is achieved [5–7]. A previous quantitative [7] and mixed method systematic review [5] described the evidence base as “emergent” [5]^ (p.411)^. Consistent reports of increased pregnancy-specific anxiety [5, 7] and preoccupation regarding the health and safety of the developing baby have been identified [5]. Internationally, there is a growing body of qualitative literature exploring pregnancy and parenting following MAC. A qualitative systematic review of seven studies conducted by Maehara et al. (2021) [8] explored the antenatal experiences of individuals who conceived via MAC. They confirmed fears regarding pregnancy loss and suggested individuals avoided developing a maternal identity to protect themselves against anticipated disappointment. Furthermore, they found changes to lifestyle in expectant mothers, which included limiting physical activity [9, 10], an increased need for reassurance [6], and often viewing themselves differently to spontaneously conceiving mothers [11, 12]. Despite this, in the UK, fertility treatment is not recognised as a risk factor for adverse perinatal mental health [13], with parents recommended to receive routine antenatal and postnatal care. Understanding the psychological, social and healthcare needs of this population is essential to ensure services appropriately meet the emotional care needs of parents following MAC.

The current literature [7, 8] focuses predominately on the antenatal experiences of the gestating partner experience. In addition, there is also evidence of hypervigilance and feelings of exclusion amongst fathers following MAC [14]. The current review is necessary to expand understanding regarding the transition to parenthood, incorporating postnatal experiences. To allow a more holistic examination of the qualitative literature, this systematic review aimed to synthesise and appraise the qualitative literature examining both parents’ experiences of pregnancy and parenthood, following MAC. This review also aimed to extract the psychological, social, and healthcare needs of parents, identifying clinical recommendations to enhance future service provision.

## Method

A systematic review and meta-synthesis, informed by the Preferred Reporting Items for Systematic reviews and Meta-Analyses guidelines [15, 16] and The Enhancing Transparency in Reporting the Synthesis of Qualitative Research (ENTREQ) [17] checklist (see Appendix 1), was conducted. The protocol was registered with PROSPERO on 2/08/2021 (REF: CRD42021269664).

### Search strategy

In developing the systematic search strategy, the Sample, Phenomenon of Interest, Design, Evaluation, Research type (SPIDER) [18] framework was initially considered. However, to ensure the search was broad and comprehensive, the final search focused on three domains of the framework only, namely Sample, Phenomenon of Interest and Research type (see Table 1). Consultation with a librarian was sought throughout the development of the search strategy.

**Table 1:** Search strategy and terms

	**Framework**	**Search terms used**
**1**	**Sample (S)**	Parent* OR Mother^a^ OR women OR Woman OR partner^a^ OR Lone paren^a^ OR same sex couple^a^ OR Same sex parent^a^ OR couple^a^ OR father^a^
**2**	**Phenomenon of Interest (PI)**	Assisted reproduction OR Medicali#ed conception OR In Vitro Fertili#ation OR IVF OR Intracytoplasmic Sperm Injection OR ICSI OR Fertility treatment OR Subfertil^a^ OR Assisted reproductive techn^a^ or Assisted reproductive treatmen^a^ or Medically assisted conception or MAC or ART
**5**	**Research type (R)**	Qualitative OR Interpretative Phenomenological Analysis OR IPA ORThematic Analysis OR Grounded Theory OR Content analysis OR Narrative^a^

The above were connected in the following way: S AND PI AND R

^a^*Truncation used to broaden search terms to include various word endings and spellings*

Two independent reviewers (ZF and ZK) conducted the literature search between February and March 2023. Electronic searches (see Appendix 2 for details) were conducted in five databases: CINAHL (EBSCO Host, 1937 to March 2023), MEDLINE (Ovid platform, 1946 to March 2023), PsycINFO (Ovid platform, 1806 to March 2023) and EMBASE (Ovid platform, 1974 to March 2023) and all editions of Web of Science (Science Citation Index Expanded 1900 – present, Social Sciences Citation Index -1900 to present, Arts & Humanitites Citation Index – 1975 to present, Conference Proceedings Citation Index – 1990 to present, Book Citation Index [BKCI] Science – 1990 to present, BKCI – Social Sciences & Humanities – 2005 to present, Emerging Sources Citation Index – 2015 to present, Current chemical Reactions – 1985 to present and Index – Chemicus – 1993 to present). Due to the multi-disciplinary nature of MAC, this database selection ensured study detection from psychological, medical and nursing literature. The databases were searched from inception to March 2023, with no restriction on publication date.

Search terms were informed by the titles and abstracts of key papers and a list of keywords and synonyms were generated. Keywords and synonyms were associated with parents, assisted reproductive treatments, qualitative research, and the antenatal and postnatal period (see Table 1). The controlled vocabulary thesaurus of each database was used when appropriate (e.g., MeSH terms and suggested subject terms) to further identify relevant papers. Truncation and Boolean operators were utilised to combine searches for individual databases.

References were exported to Endnote Reference Managing software (Clarivate Analytics UK Ltd [version 20], 2020) and duplicates removed using the ‘remove duplicates’ function. Database searches were supplemented by forward and backward searching the reference lists of included studies. The titles and abstracts of all obtained articles were screened independently against the inclusion criteria by the first author and another reviewer (ZK), who was not part of the author team. Full text versions of retained articles were read by two researchers independently (ZF and ZK) and were assessed for eligibility. All authors were involved in the final decision of which papers to include in this review.

### Inclusion criteria for studies

Studies were included if they 1) were an empirical study that used qualitative methods to explore and analyse gestating individuals and/or their partners’ experiences of pregnancy and parenting following successful MAC (i.e., studies were included even if only one parent reported their experiences), 2) were published in a peer-reviewed journal, and 3) focused on MAC treatments, such as IVF, ICSI, Gamete Intrafallopian Transfer and the use of donor gametes. Those that specifically focused on Intrauterine Insemination (IUI), or Ovulation Induction (OI) were excluded due to their less invasive nature. However, mixed samples of MAC and IUI or OI were retained. Studies meeting the inclusion criteria published in any language were included and translated when possible.

To minimise the inclusion of studies lacking peer review and potentially conducted with less standardised scientific rigour, grey literature was excluded. Studies were also excluded when they deviated from the experience of MAC itself and focused on specific topics, such as fertility, use of donor gametes or surrogacy and when they did not focus on the parents’ perspective or lived experiences, for example, only reporting professional views.

### Methodological quality and risk of bias assessment

All studies were assessed for methodological quality using the Critical Appraisal Skills Programme (CASP) [19] qualitative checklist. The CASP is a widely employed quality assessment tool in qualitative health-related synthesis [20] and includes questions relating to study validity, design and results. A numerical value was prescribed to checklist items (No = 0, Can’t Tell = 0.5, Yes = 1) to generate a useful indicator for comparison, an approach adopted in a previous meta-synthesis [21]. The total CASP score for each paper was then categorised as either ‘high’ (> 8–10), ‘moderate’ (6–8) or ‘low’ ($\le$ 5) quality. The first author conducted the quality assessment of all included papers. To assess reliability, an independent reviewer rated 50% of the included papers. Both parties independently reviewed the randomly selected papers, writing supplementary notes to support the decision, which were reviewed collaboratively. The latter was fundamental, helping to ground the decision in evidence. Any disagreements regarding rating were discussed and resolved through discussion and returning to the original articles.

### Data extraction and synthesis approach

Relevant data (e.g., the author(s), location of the study, year of publication, study aims, sample characteristics, the type of MAC participants had experienced, data analysis method and results) were extracted from the studies and tabulated chronologically according to antenatal experience, postnatal experience or studies covering both. A thematic synthesis was adopted allowing for new interpretation of multiple findings and the development of analytical themes, furthering understanding [22, 23]. A critical realist perspective position was adopted as the epistemological stance [24] due to its focus on understanding rather than describing phenomena. Central to the critical realist position is integrating ontology (typical questions include “what is real/independent of our perceptions?”) with constructivist epistemology [25]. This epistemological stance acknowledges that independent of theory, psychosocial processes exist; however, meaning can be constructed from the included studies. This stance also allowed inferences to be made about the psychosocial processes linked to pregnancy and parenting following MAC, whilst recognising that these inferences are subjective and embedded within the context of the research. During data analysis, the authors were aware of their own positions and reflexivity.

All text under the original papers’ ‘Results’ or ‘Findings’ headings were extracted into NVivo software (QSR International Pty Ltd., 2020). The first author undertook the stages of thematic synthesis. This included line-by-line coding, the development of descriptive themes and analytical themes. Firstly, each line of the primary study’s findings was coded according to meaning and content. Following this, studies were coded into pre-existing codes and new codes created when required. The first author looked for similarities and differences between the codes and they were grouped into related areas to develop descriptive themes. Codes were identified within and across studies and collated based on coherence. Analytical themes were then developed inductively by synthesising the findings across studies, interpreting their meaning. An experienced qualitative post-doctoral researcher scrutinised the data to ensure codes and themes were appropriately derived from the data and were acceptable. All themes and subthemes were discussed, refined and agreed by all authors.

### Reflexivity statement

Reflexivity is a central component of qualitative research because the researchers’ assumptions, beliefs and prior experiences, both personal and professional, influence the research process. Acknowledgement of the researchers’ reflexive position enhances rigour, credibility and extends understanding of the findings [26]. The academic research team comprised four white British and/or European women who had a wealth of clinical, academic and lived experience. All research team members had a firm philosophy in patient-centred, preventative care and a shared interest in understanding the psychological factors impacting expectant and new parents. The first author was a practising Trainee Clinical Psychologist who had experience of working antenatally and postnatally with expectant and new parents experiencing relational difficulties. DS was an experienced Health Psychologist whereas AW was a Clinical Psychologist and researcher, with particular interests in parenting and maternal mental health during the perinatal period. LH was an expert, working clinically and in a research capacity, in the field of Obstetrics, bringing a medical perspective. All members of the research team were mothers with varying experiences of conception, including IVF, pregnancy and loss.

The first author kept a reflective log throughout the research process. As part of the data analysis process, interpretations were shared between the first author and DS, challenging each other’s pre-conceptions, allowing new meaning to be distilled. Furthermore, the research team regularly discussed the interpretation of data, reflecting on our positioning, challenging our own biases and beliefs.

## Results

### Study characteristics

**Fig. 1:**
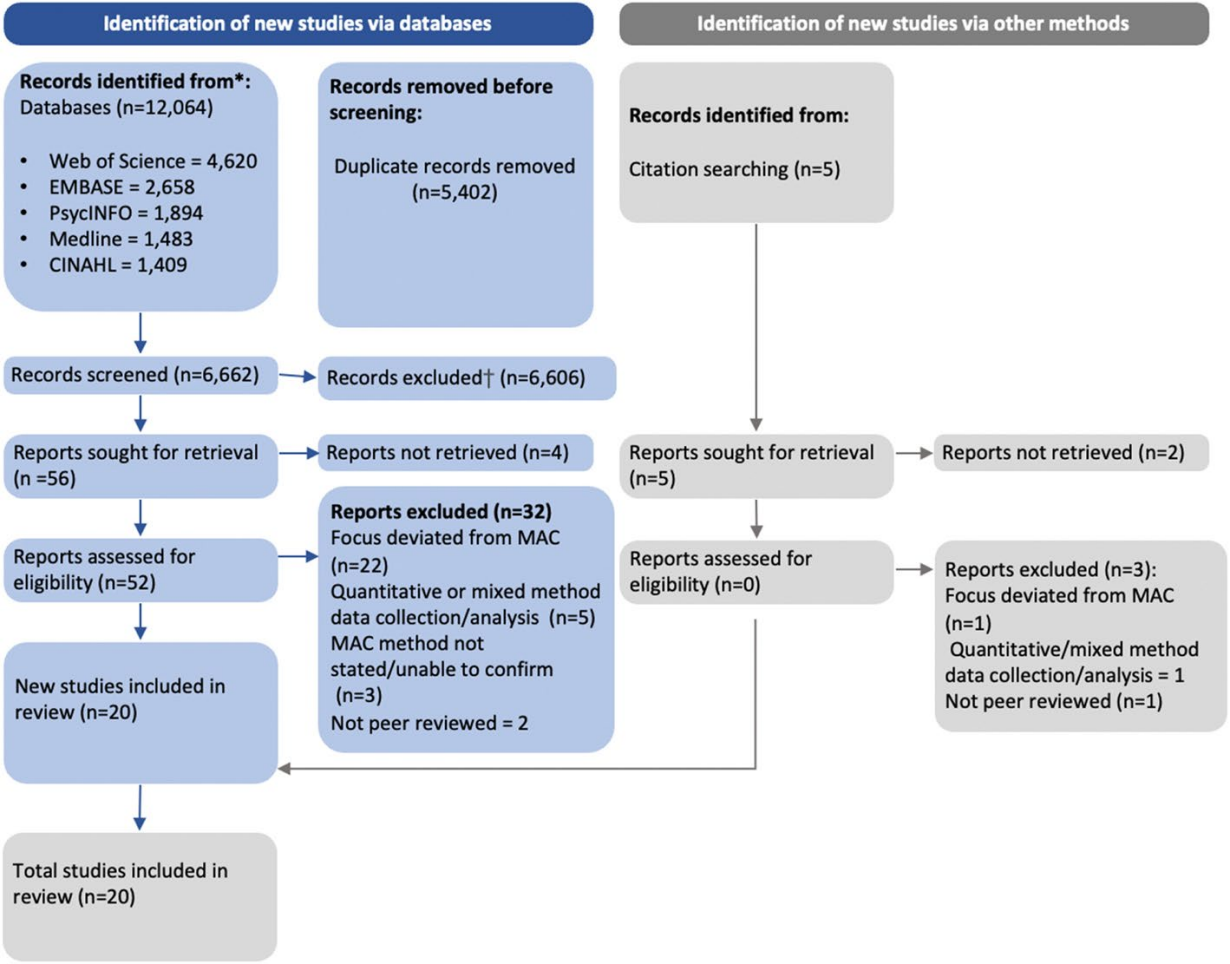
Prisma flow diagram outlining the systematic process

The electronic search identified 6,662 articles and another five articles were identified by forward and backward searching. Following the independent screening of 6,667 articles by two reviewers, 6,611 papers were excluded. After the eligibility check of retained articles, a total of 20 qualitative studies, drawing on 19 participant samples, were included in the review (see Fig. 1).

These studies represented the experiences of 300 individuals. Most studies were conducted in Asia (*n* = 7) and Europe (*n* = 7) followed by South America (*n* = 5), and Australia (*n* = 1). Six studies were translated to English using an online service (www.onlinedoctranslator.com) and checked for accuracy by native speakers. The average sample size was 15, ranging from 3 to 51 participants. The studies captured the views of mothers/expectant mothers (*n* = 13), fathers/expectant fathers (*n* = 2) and couples (*n* = 5), antenatally (*n* = 12), postnatally (*n* = 4) or their experiences of both (*n* = 4). Many studies explored participants’ views and experiences of pregnancy and parenting more generally (*n* = 16). Some studies focussed on breastfeeding (*n* = 1), the couple’s relationship (*n* = 1), physical activity (*n* = 1) and the midwifery care needs of participants (*n* = 1). Table 2 summarises the study characteristics.

**Table 2:** Overview of all 20 included studies

	**Authors and location**	**Aims**	**Data collection**	**Participant demographics**	**MAC detail**	**Data analysis**	**Main themes**
**Studies exploring the antenatal period and birth**							
** 1**	Dornelles, MacCallum, Lopes, Piccinini and Passos (2016) [27] Brazil	To understand the perceptions of pregnancy achieved after ART Whether the above experience is affected by previous failed treatment cycles	Semi-structured interviews (Length approximately 90 min)	19 expectant first-time mothers in the third trimester of pregnancy **Demographics** **Mean age** 35 years (range 25–44) **Ethnicity** White = 17 (89%|) **Marital status** Married = 12 (63%) Cohabiting = 7 (37%) **Education** University degree = 12 (63%) High school = 7 (37%) **Pregnancy** Singleton = 15 (79%) Twins = 1(5%) Triplets = 3 (16%)	**MAC indication** Female = 15 (79%) Male = 2 (10.5%) Combined = 1 (5.3%) Unexplained = 1 (5.3%) **MAC type** IVF = 15 (79%) OI = 0 (0%) IUI = 3 (16%) Not specified^a^ = 1 (5%) **MAC treatment history** First attempt = 11 (57%) Two or more attempts = 8 (43%)	Thematic analysis (Laville & Dionne 1999) [28]	Three main themes: 1) Tolerance of the demands of treatment/pregnancy 2) Consideration of the mechanics of treatment and pregnancy 3) Emotionally painful aspects of treatment/pregnancy
** 2**	Sonego., Dornelles, Lopes, Piccinini and Passos (2017) [14] Brazil	To investigate the experience of pregnancy after ART from the father’s perspective	Semi-structured interview using the *Pregnancy and Expectations of the Future Father* (NUDIF, 1998b (Length approximately 90 min)	13 men whose partners were in the 3^rd^ trimester of pregnancy **Demographics** **Mean age** Not reported (range = 32–46 years) **Ethnicity** Not reported **Marital status** Married or cohabiting = 13 (100%) **Education** Elementary school = 2 (15%) High school = 4 (31%) Incomplete higher education = 1 (8%) Completed higher education = 6 (46%) **Pregnancy** Single = 10 (77%) Multiple = 3 (23%)	**MAC indication** Female = 9 (69%) Male = 3 (23%) Combined = 1 (8%) Unexplained = 0 (0%) **MAC type** IVF = 9 (70%) OI = 0 (0%) IUI = 2 (15%) Not specified^a^ = 2 (15%) **MAC treatment history** First attempt = 7 (54%) Two or more attempts^b^ = 6 (46%)	Content analysis (Laville & Dionne, 1999) [28]	Two main themes: 1) Subjective experience of the father during pregnancy 2) Effects of treatment on the experience of pregnancy
** 3**	Walker, Mills and Gilchrist (2017) [10] UK	To explore the qualitative experiences and decision-making processes regarding physical activity in women who have undergone IVF	Individual semi-structured interviews (Length not reported)	8 women who had successfully conceived via IVF/ICSCI in the last two years **Demographics** **Mean age** 33 years old (range = 24–39) **Ethnicity** White British = 8 (100%) **Marital status** Not reported **Education** Not reported **Pregnancy** Not reported	**MAC indication** Not reported **MAC type** IVF = 8 (100%) OI = 0 (0%) IUI = 0 (0%) Not specified = 0 (0%) **MAC treatment history** Not reported	Interpretative phenomenological analysis (IPA; Smith, Flowers & Larkin, 2009) [29]	Three main themes 1) Navigating away from childlessness and towards motherhood 2) Negotiating a safe passage 3) Balancing the challenges of pregnancy with the needs of the self
** 4**	Warmelink, Adema, Pranger and Paul de Cock (2016) [30] Netherlands	To investigate the midwifery care needs during pregnancy of couples or women who have conceived as a result of fertility treatment	Semi-structured, in-depth interviews (Average length 55 min. Ranged from 28–91 min)	9 interviews with 11 participants (two couples: Female = 9; Male = 2) **Demographics** **Mean age** Female mean age = 34.1 years (range = 32–38 years) Male mean age = 33.5 years (range = 32 -35 years) **Ethnicity** Dutch = 100% **Marital status** Not reported **Educatio**n Not reported **Pregnancy** Not reported	**MAC indication** Female = 2 (22%) Male = 4 (44%) Combined = 1 (11%) Unexplained = 2 (22%) **MAC type** IVF = 10 (91%) OI = 0 (0%) IUI = 1 (9%) **MAC treatment history** Not reported	Content analysis (Boeije, 2008) [31]	Four main themes: 1) Normal but not normal: Paradoxical feelings 2) Understanding the impact of previous history 3) Psychosocial support 4) Care needs in general
** 5**	French, Sharp and Turner (2015) [12] UK	To explore the antenatal experiences of males and females who have successfully conceived via fertility treatment	Individual interviews (Length approximately 1–3 h)	20 participants 12 females; 8 males interviewed at 28 weeks’ gestation of first pregnancy **Demographics** **Mean age** Not reported (Age range = 35–39 years) **Ethnicity** Not reported **Marital status** Not reported **Education** Not reported **Pregnancy** Not reported	**MAC indication** Female = 7 (35%) Male = 6 (30%) Combined = 2 (10%) Unexplained = 5 (25%) **MAC type** IVF = 17 (85%) OI = 2 (10%) IUI = 1 (5%) **MAC treatment history** Not reported	Thematic analysis- constant comparison (Patton, 1990) [32]	Four main themes: 1) Fear of pregnancy loss 2) Difficulty adjusting to pregnancy and planning for parenthood 3) Gaps in care 4) Self-silencing
** 6**	Ranjbar, Akhondi, Borimnejad, Ghaffari and Behboodi-Moghadam (2015) [9] Iran	To explore how women make sense of assisted pregnancy in Iranian culture and context	Semi-structured, interviews (Length approximately 30–60 min)	12 women who experiences assisted pregnancy with their first child **Demographics** **Mean age** 29.51 (Age range = 24–36) **Ethnicity** Iranian Kurd = 2 (17%) Fars = 6 (50%) Turk = 3 (25%) Lor = 1 (8%) **Marital status** 100% married **Education** MSc/MA = 2 (16.7%) BS = 3 (25%) High School = 6 (50%) 9^th^ grade = 1 (8%) **Pregnancy** Single = 12	**MAC indication** Female = 12 (100%) Male = 0 (0%) Combined = 0 (0%) Unexplained = 0 (0%) **MAC type** IVF = 12 (100%) OI = 0 (0%) IUI = 0 (0%) Not specified = 0 (0%) **MAC treatment history** Not reported	Interpretative phenomenological approach (Van Manen, 1990) [33]	Three main themes: 1) Finding peace in life 2) Paradoxical feelings 3) Struggling to realise a dream
** 7**	Dornelles, MacCallum, Lopes, Piccinini and Passos (2014) [34] Brazil	To explore women’s fears during pregnancy following conception via assisted reproductive technology (ART)	Semi-structured interviews (Length approximately 90 min)	19 first time mothers in their third trimester of pregnancy **Demographics** **Mean age** 35 years (range 25–44) **Ethnicity** White = 17 (89%) Other ethnicity = 2 (11%) **Marital status** Married = 12 (63%) Cohabiting = 7 (37%) **Education** University degree = 12 (63%) High School graduates = 7 (37%) **Pregnancy** Single pregnancy = 15 (80%) Twins = 1 (5%) Triplets = 3 (15%)	**MAC indication** Female = 15 (79%) Male = 2 (11%) Combined = 1 (5%) Unexplained = 1 (5%) **MAC type** IVF = 15 (79%) OI = 0 (0%) IUI = 3 (16%) Not specified^a^ = 1(5%) **MAC treatment history** First attempt = 10 (53%) Two or more attempts = 9 (47%)	Content analysis (Laville & Dionne, 1999) [28]	Four main themes: 1) The baby’s survival 2) The health of the baby 3) The efficacy of the other 4) Childbirth
** 8**	Lin, Tsai and Lai (2013) [35] Taiwan	To describe the experiences of pregnancy in Taiwanese women who had undergone at least three cycles of ART over a period exceeding three years	In-depth interviews (Length approximately 90–120 min)	15 females interviewed within one year of delivering a baby **Demographics** **Mean age** 39 years (range 31–44) **Ethnicity** Not reported **Marital status** Married = 15 (100%) **Education** MSc = 5 (33%) Bachelor’s degree = 8 (53%) Junior college degree = 1 (7%) High School Diploma = 1 (7%) **Pregnancy** Not reported	**MAC indication** Female factor = 15 (100%) Male factor = 0 (0%) Combined = 0 (0%) Unexplained = 0 (0%) **MAC type** IVF = 15 (100%) OI = 0 (0%) IUI = 0 (0%) Not specified = 0 (0%) **MAC treatment history** First attempt = 0 (0%) Two or more attempts = 15 (100%)	Phenomenological qualitative method procedures adopted by Creswell (2009) [25]	Five main themes: 1) Emphasis on the safety and health of the foetus 2) Psychosocial reactions to physical and physiological conditions 3) Transition of identity 4) Insights after going through pregnancy and labour 5) Impact of society on pregnancy
** 9**	Smorti and Smorti (2013) [36] Italy	To explore the psychological processes that develop in women and men during their first pregnancy obtained with assisted reproduction treatment	Semi-structured autobiographical interview (Length approximately (50–90 min)	15 Italian couples pregnant with their first child (29^th^-34 weeks pregnant) **Demographics** **Mean age** Female = 36 years Male = 38 years **Ethnicity** White = 14 (93%) Other ethnicity = 1 (7%) **Marital status** Married or cohabiting = 15 (100%) **Education** Women degree or high school = 13 (87%) Men completed secondary school = 13 (86%) P**regnancy** Single = 12 (80%) Twins = 2 (13%) Multiple pregnancy = 1 (7%)	**MAC indication** Female = 6 (40%) Male = 5 (36%) Combined = 1 (4%) Unexplained = 3 (20%) **MAC type** IVF = 7 (48%) OI = 0 (0%) IUI = 8 (52%) Not specified = 0 (0%) **MAC treatment history** First attempt = 6 (40%) Two or more attempts = 9 (60%)	Identification of themes, patterns on global and qualitative level	Participants narrated their pregnancy experience as a process with four main phases: 1^st^ phase: ‘Doubt’ phase 2^nd^ phase: Anxious and overwhelming need to seek help and support re: fertility 3^rd^ phase: struggle and the victory 4^th^ phase: The monitoring phase
** 10**	Dornelles and Lopes (2011) [37] Brazil	To understand the process of becoming a mother in the context of ART	Interview on Pregnancy and Expectations of the Pregnant women (NUDIF, 1998b) (Length not reported)	3 participants in their third trimester of pregnancy with their first child **Mean age** 35 years (range = 25–37) **Ethnicity** Not reported **Marital status** Not reported **Education:** Higher education: 2 (67%) Middle education: 1 (33%) **Pregnancy** Not reported	**MAC indication** Female = 1 (33%) Male = 1 (33%) Combined = 0 (0%) Unexplained = 1 (33%) **MAC type** IVF = 1 (33%) OI = 0 (0%) IUI = 1 (33%) Not specified^a^ = 1 (33%) **MAC treatment history** First attempt = 1 (33%) Two or more attempts = 2 (66%)	Content analysis (Laville & Dionne, 1999) [28]	Six main themes: 1) Life growth theme 2) Theme relating to primary 3) Support matrix – 4) Identity reorganisation – 5) Stages of conception: pregnancy 6) Imaginary baby
** 11**	Silva and Lopes (2011) [38] Brazil	To investigate the marital relationship during treatment and pregnancy in couples who became pregnant with the help of ART	Semi —structured interviews (Length approximately 120 min)	6 participants (three couples) **Mean age and range** Not reported **Ethnicity** Not reported **Marital status** In a relationship = 6 (100%) **Education** Higher education = 4 (67%) Elementary school = 2 (33%) **Pregnancy status** Not reported	**MAC indication** Female = 2 (33%) Male = 1 (33%) Combined = 0 (0%) Unexplained = 0 (0%) **MAC type** IVF = 2 (66%) OI = 0 (0%) IUI = 0 (0%) Not specified^a^ = 1 (33%) **MAC treatment history** First attempts = 1 (33%) Two or more attempts = 2 (66%)	Content analysis (Laville & Dione, 1999) [28]	Six main themes: 1) Cohesion during treatment 2) Cohesion during pregnancy 3) Sexuality during treatment 4) Sexuality during pregnancy 5) Communication during treatment 6) Communication during pregnancy
12	Hayashi and Sayama (2009) [11] Japan	To qualitatively highlight the emotional processes experienced by women who achieved pregnancy via assisted reproductive technology	Semi-structured interviews (Average length = 64.5 min; range = 46–87 min)	8 primiparas who achieved pregnancy via ART one to six months after delivery **Demographics** **Mean age** 34 years (age range = 28–42) **Ethnicity** Not reported **Marital status** Not reported **Education status** Not reported **Pregnancy status** Single = 6 (75%) Twins = 2 (25%)	**MAC indication** Not reported **MAC type** IVF = 7 (88%) OI = 0 (0%) IUI = 0 (0%) Other = 1 (12%) **MAC treatment history** Not reported	Phenomenological study method (Colazzi, 1978) [39]	Nine main themes: 1) Feelings of mission and pressure by becoming pregnant 2) Attention to the avoided jealousy 3) Wisdom in overcoming anxiety 4) Maternal self-consciousness 5) Release from feelings of loneliness 6) Recovery of self-confidence 7) Positive acceptance of infertility and the treatment experience 8) Confirming one’s own growth 9) Feeling authentic joy from pregnancy
**Studies exploring birth and/or postpartum experiences**							
** 13**	Díaz Sáez, Fernandez-Medina, Granero-Molina, Fernandez-Sola, Hernandez-Padilla and Lopez-Rodrugues (2021) [40] Spain	To describe and understand the breastfeeding experiences of first-time mothers who conceived using ART	Focus group (*n* = 8) lasting 86 min and Individual semi-structured interviews (*n* = 19) (Average length = 37 min.)	27 women first time mothers **Demographics** **Mean age** 38 years (age range = not reported) **Ethnicity** Not reported **Marital status** Married = 21 (78%) Cohabiting = 6 (22%) **Education** Higher Education = 15 (56%) Medium = 2 (7%) Basic = 10 (37%) **Pregnancy** Single = 27 (100%)	**MAC indication** Not reported **MAC type** IVF = 16 (59%) OI = 0 (0%) IUI = 11 (41%) Not specified = 0 (0%) **MAC treatment history** Not reported	Hermeneutic phenomenology (Gadamer, 2005) [41]	Two main themes: 1) The transition from infertility to motherhood 2) The reality of becoming a breastfeeding mother after ART
** 14**	Sadeghi, Mohammadi, Mohammadpourand and Abbasi (2019) [42] Iran	To investigate the challenges mothers face after assisted-reproduction techniques Part of a larger phenomenological study that aimed to discover the experience of motherhood after ART	Sem- structured interviews, (Length not report)	13 mothers who conceived via ART **Demographics** **Mean age** 32 years **Ethnicity** Not reported **Marital status** Not Reported **Education** Diploma = 7 (54%) Bachelor = 6 (46%) **Pregnancy** Not reported	**MAC indication** Female = 8 (62%) Male = 5 (38%) Combined = 0 (0%) Unexplained = 0 (0%) **MAC type** IVF = 9 (69%) OI = 0 (0%) IUI = 3 (31%) Not specified = 0 (0%) **MAC treatment history** Not reported	Hermeneutic phenomenological method incorporating thematic analysis (Van Manen, 1990) [33]	One main theme: 1) ‘Over-challenged mother’
** 15**	Mohammadi, Shamshiri, Mohammadpour, Vehilainen-Julkunen, Abbasi and Sadeghi (2015) [43] Iran	To explore and describe the experience and meaning of mothering after ART among Iranian women	Semi structured interviews (Length approximately 45–70 min)	9 first time mothers **Demographics** **Mean age** 32 years (age range = 28–45) **Ethnicity** Not report **Marital status** Not reported **Education** Diploma = 6 (67%) Bachelor = 3 (33%) **Pregnancy** Not reported	**MAC indication** Female = 6 (67%) Male = 3 (33%) Combined = 0 (0%) Unexplained = 0 (0%) **MAC type** IVF = 6 (67%) OI = 0 (0%) IUI = 3 (33%) Not specified = 0 (0%) **MAC treatment history** Not reported	Heideggerian hermeneutic phenomenological approach (cited in Van Manen, 1990) [33]	One main theme: 1) ‘Super-mothering’
** 16**	Bracks- Zalloua, McMahon and Gibson (2011) [44] Australia	To provide an in-depth understanding of early parenthood for IVF-conceiving fathers	Semi-structured interviews (Length approximately one hour)	8 men whose partners had conceived via IVF **Demographics** **Mean age** 40 years old (range 29–53 years) **Marital status** Married = 7 (88%) Cohabiting = 1 (12%) **Ethnicity** Western = 8 (100%) **Pregnancy** Not reported	**MAC indication** Female = 4 (50%) Male = 4 (50%) Combined = 0 (0%) Unexplained = 0 (0%) **MAC type** IVF = 8 (100%) OI = 0 (0%) IUI = 0 (0%) Not specified = 0 (0%) **MAC treatment history** Not reported	Modified analytic induction (Bogdan & Biklem, 1998) [45]	Three main themes: 1) The concerned partner 2) Inattention from partner 3) Interaction with child
**Studies exploring the antenatal and postnatal period**							
** 17**	Boz, Teskereci and Akgun (2021) [46] Turkey	The experience of becoming a mother following successful IVF: a grounded theory	Semi-structured interviews (length not reported)	18 mothers who had become pregnant and had a child following successful IVF **Demographics** **Mean age** 32 years (age range = 27–39) **Ethnicity** Not reported **Marital status** Not reported **Education** Higher education = 6 (33%) High school = 2 (11%) Secondary school = 8 (44%) Vocational school = 2 (11%) **Pregnancy** Not reported	**MAC indication** Female = 2 (11%) Male = 6 (33%) Combined = 1 (6%) Unexplained = 9 (50%) **MAC type** IVF = 18 (100%) OI = 0 (0%) IUI = 0 (0%) Not specified = 0 (0%) **MAC treatment history** First attempt = 16 (89%) Two or more attempts = 2 (11%)	Grounded Theory (Charmaz, 2014) [47]	Four themes: 1) Non-spontaneous path to motherhood a) treatment 2) Leaving the infertility world 3) Pregnancy under the shadow of fear 4) Getting stuck between fertile and infertile worlds
** 18**	Allan, Mounce, Cullem Van den Akket and Hudson (2019) [48] UK	To explore non-donor IVF couples’ transition to early parenthood	Unstructured interviews with couples (length approximately 40–60 min)	16 heterosexual couples with one live singleton infant **Demographics** **Mean age** Not reported (Female age range = 25–39) (Male age range = 29–41) **Ethnicity** Not reported **Marital status** Not reported **Education** Not reported **Pregnancy** Single = 16 (100%)	**MAC indication** Not reported **MAC type** IVF = 16 (100%) OI = 0 (0%) IUI = 0 (0%) Not specified = 0 (0%) **MAC treatment history** First attempt = 9 (56%) Two or more attempts = 6 (38%) Unknown = 1 (6%)	Thematically (Frost, 2010) [49]	Three themes: 1) Preparing for parenthood 2) Becoming a parent 3) Considering a sibling
** 19**	Crespo and Bestard (2016) [50] Spain	To explore the psychosocial needs of women and their partners following assisted reproductive treatment in a Spanish Context	Repeated rounds of semi-structured interviews (Length approximately 30–90 min)	51 participants (30 pregnant women; 21 partners) **Demographics** **Mean age** 37 years (age range = not reported) **Ethnicity** Spanish = 51 (100%) **Marital status** Married = 25 (83%) Single = 4 (13%) Divorced = 1 (3%) **Education** Higher education = 17 (57%) Secondary = 9 (30%) Primary = 4 (13%) **Pregnancy** Foetus reduction = 6 (50%) Single pregnancy =	**MAC indication** Female – not reported Male – not reported Combined – not reported Unexplained – not reported Same-sex couple = 1 (3%) One lone parent = 1 (3%) **MAC type** IVF = 26 (87%) OI = 0 (0%) IUI = 4 (13%) **MAC treatment history** Not reported	Does not state – text was coded into either predetermined or emergent topics	Two major themes: 1) Complexity of reasons for anxiety 2) Narrowing experience
** 20**	Katsumara, Kamiya and Emisu (2014) [51] Japan	To clarify the experiences from pregnancy with a first child to puerperiuem and childcare, of women who became pregnant through fertility treatment	Semi-structured interviews (length approximately 29–60 min)	9 women who received fertility treatment for a second pregnancy after giving birth to a first child through fertility treatment **Mean age** 37 years old (range = 33–43 years old) **Ethnicity** Not reported **Marital status** Not reported **Education** Not reported **Pregnancy** Not reported	**MAC indication** Female = 4 (44%) Male = 1 (12%) Combined = 0 (0%) Unexplained = 4 (44%) **MAC type** IVF = 6 (67%) OI = 0 (0%) IUI = 2 (23%) **MAC treatment history** Not reported	Content analysis (Greg et al., 2007) [52]	17 main themes: 1) Growing desire for a second child; contrary to expectations 2) Lack of actual sense of pregnancy and delivery 3) Joy and pride in pregnancy 4) Anxiety and reassurance in selecting a birthing facility 5) Feeling of relief at having to come this far on a long journey 6) Acceptance that one can not have a natural delivery 7) Fluctuation between anxiety and abnormalities or disorders in the fetus and feeling that it will be all right 8) Thankfulness and stress with respect to family 9) Uncertainty about continuation of pregnancy 10) Longing for the birth of a healthy child 11) Delivery with little sense of fulfilment 12) Joy at becoming a mother and motivation for child rearing 13) Joy felt from existence of baby 14) Easing of worries regarding baby 15) Anxiety about raising one’s first child despite joyful birth after much difficulty 16) Weakness of one’s feelings and emotion towards birth 17) Connection between unsettling events and medical treatment

*Abbreviations*: *IV*F In-Vitro Fertilisation, *OI* Ovulation Induction, *IUI* Intrauterine Insemination

^a^ Gamete donation – unknown whether this was sperm or egg donation

The most reported demographic information was age (*n* = 19, 95%), followed by education (*n* = 12, 60%), marital status (*n* = 8, 40%) and ethnicity (*n* = 8, 40%). Participants ranged from 24 to 53 years in age and were commonly educated to degree level (see Table 2). Various MAC methods were recorded across studies, including IVF (*n* = 18, 90%), ICSI (*n* = 7, 35%) and the use of donor gametes (*n* = 6, 30%). Eleven studies (55%) had mixed samples of MAC and other less invasive forms of treatment, such as IUI or OI.

Most studies (*n* = 17, 85%) recruited individuals with a history of infertility; only one study (5%) recruited a mixed sample including a same-sex couple and a lone parent [26]. Three studies (15%) did not report the reason for MAC [10, 40, 48].

All studies collected data via interview, ranging in length from 29 to 180 min. The qualitative methodology most frequently employed was Interpretative Phenomenology (*n* = 7, 35%), followed by Content Analysis (*n* = 6, 30%), Thematic Analysis (*n* = 3, 15%), Grounded Theory (*n* = 1, 5%) and Modified Analytic Induction (*n* = 1, 5%). Two studies (10%) did not specify their method [36, 50].

### Quality appraisal and risk of bias of studies

Methodological quality varied across studies, as can be seen in Table 3. Agreement between raters was 94% (k = 0.765, *p* < 0.001, rated as ‘substantial agreement’). Most studies were rated as ‘moderate’ quality (*n* = 13), followed by high (*n* = 5). Two studies [37, 51] were rated low in quality. To ensure comprehensiveness, papers of low quality were retained; however, it was necessary to report the quality ratings for each study for transparency. No studies sufficiently referenced reflexivity, a fundamental process to qualitative research; thus, this was a general weakness of all studies.

**Table 3:** Overview of the methodological quality appraisal of the 20 included studies

	**Study**	**Aims**	**Methodology**	**Design**	**Recruitment**	**Data collection**	**Reflexivity**	**Ethical issues**	**Data analysis**	**Statement of findings**	**Valuable**	**Score and overall rating (0–10)**
1	Dornelles et al. (2016) [27]	Yes (1)	Yes (1)	No (0)	C T (0.5)	Yes (1)	No (0)	Yes (1)	C T (0.5)	Yes (1)	Yes (1)	**Moderate** **(7)**
2	Sonego et al. (2017)[14]	Yes (1)	Yes (1)	No (0)	C T (0.5)	Yes (1)	No (0)	No (0)	C T (0.5)	Yes (1)	Yes (1)	**Moderate** **(6)**
3	Walker et al. (2017) [10]	Yes (1)	Yes (1)	Yes (1)	No (0)	C T (0.5)	C T (0.5)	C T (0.5)	C T (0.5)	Yes (1)	Yes (1)	**Moderate** **(7)**
4	Warmelink et al. (2016) [30]	Yes (1)	Yes (1)	C T (0.5)	C T (0.5)	C T (0.5)	N (0)	C T (0.5)	Yes (1)	Yes (1)	Yes (1)	**Moderate** **(7)**
5	French et al. (2015) [12]	Yes (1)	Yes (1)	C T (0.5)	Yes (1)	Yes (1)	No (0)	C T (0.5)	Yes (1)	Yes (1)	Yes (1)	**High** **(8)**
6	Ranjbar et al. (2015) [9]	Yes (1)	Yes (1)	C T (0.5)	No (0)	C T (0.5)	No (0)	Yes (1)	Yes (1)	Yes (1)	Yes (1)	**Moderate** **(7)**
7	Dornelles et al. (2014) [34]	Yes (1)	Yes (1)	No (0)	C T (0.5)	C T (0.5)	No (0)	Yes (1)	No (0)	Yes (1)	Yes (1)	**Moderate** **(6)**
8	Lin et al. (2013) [35]	Yes (1)	Yes (1)	Yes (1)	Yes (1)	Yes (1)	No (0)	C T (0.5)	Yes (1)	Yes (1)	Yes (1)	**High** **(8.5)**
9	Smorti and Smorti (2013) [36]	Yes (1)	Yes (1)	Yes (1)	Yes (1)	Yes (1)	No (0)	C T (0.5)	No (0)	Yes (1)	Yes (1)	**Moderate** **(7.5)**
10	Dornelles and Lopes (2011) [37]	Yes (1)	Yes (1)	C T (0.5)	C T (0.5)	No (0)	No (0)	C T (0.5)	No (0)	Yes (1)	No (0)	**Low** **(4.5)**
11	Silva and Lopes (2011) [38]	Yes (1)	Yes (1)	No (0)	Yes (1)	C T (0.5)	No (0)	Yes (1)	No (0)	Yes (1)	C T (0.5)	**Moderate** **(6)**
12	Hayashi and Sayama (2009) [11]	Yes (1)	Yes (1)	Yes (1)	C T (0.5)	Yes (1)	No (0)	Yes (1)	Yes (1)	Yes (1)	No (0)	**Moderate** **(7.5)**
13	Díaz Sáez et al. (2021) [40]	Yes (1)	Yes (1)	Yes (1)	C T (0.5)	C T (0.5)	No (0)	Yes (1)	C T (0.5)	Yes (1)	Yes (1)	**Moderate** **(7)**
14	Sadeghi et al. (2019) [42]	Yes (1)	Yes (1)	Yes (1)	C T (0.5)	Yes (1)	C T (0.5)	Yes (1)	C T (0.5)	Yes (1)	Yes (1)	**Moderate** **(7.5)**
15	Mohammadi et al. (2015) [43]	Yes (1)	Yes (1)	Yes (1)	Yes (1)	Yes (1)	No (0)	Yes (1)	C T (0.5)	Yes (1)	Yes (1)	**High** **(8.5)**
16	Bracks-Zalloua et al. (2011) [44]	Yes (1)	Yes (1)	Yes (1)	Yes (1)	Yes (1)	No (0)	No (0)	Yes (1)	Yes (1)	Yes (1)	**High** **(8)**
17	Boz et al. (2021) [46]	Yes (1)	Yes (1)	Yes (1)	Yes (1)	Yes (1)	No (0)	Yes (1)	Yes (1)	Yes (1)	Yes (1)	**High** **(9)**
18	Allan et al. (2019) [48]	Yes (1)	Yes (1)	C T (0.5)	C T (0.5)	Yes (1)	No (0)	Yes (1)	No (0)	Yes (1)	Yes (1)	**Moderate** **(7)**
19	Crespo and Bestard (2016) [50]	Yes (1)	Yes (1)	Yes (1)	C T (0.5)	Yes (1)	No (0)	C T (0.5)	C T (0.5)	C T (0.5)	Yes (1)	**Moderate** **(7)** **e**
20	Katsumara et al. (2014) [51]	Yes (1)	Yes (1)	C T (0.5)	No (0)	C T (0.5)	No (0)	Yes (1)	No (0)	No (0)	C T (0.5)	**Low** **(4.5)**
	% of included studies rated as ‘Yes’	100%	100%	50%	35%	60%	0%	55%	35%	90%	80%	

*Abbreviation: CT Can’t Tell*

### Findings

**Fig. 2:**
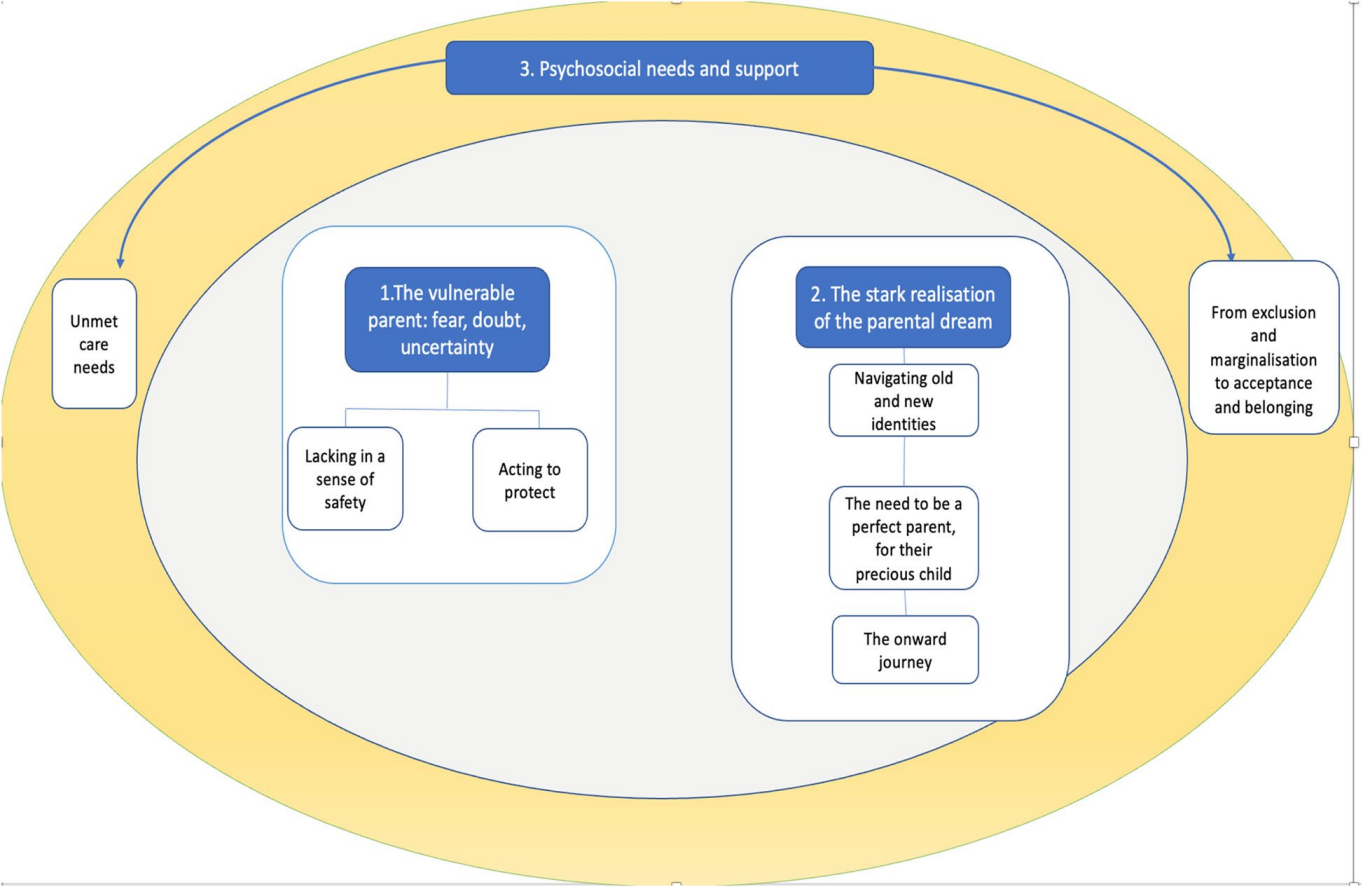
Conceptual map of identified main themes and subthemes

Three main themes and seven subthemes were identified (Fig. 2), which will be illustrated through selected quotes. A matrix of themes (see Appendix 3) illustrates which themes were present for each study.

### Theme 1: The vulnerable parent: fear, doubt and uncertainty

This theme, consisting of two subthemes, encompassed the uncertainty, doubt and fear individuals experienced as they entered pregnancy, lacking a sense of safety. Commonly, this lack of safety was expressed as hypervigilance, fears of pregnancy loss and fetal abnormalities, with parents living cautiously, seeking reassurance and acting to protect their child during pregnancy and parenting.

#### Subtheme 1.1: Lacking a sense of safety

Pregnancy was an emotionally overwhelming time for expectant parents. Ambivalence often accompanied successful conception, with paradoxical feelings reported [9, 46, 50, 51]. Some parents described it as “the end of a nightmare” [36]^ (p.171)^, feeling victorious that their “fight” [36] ^(p.171)^ with fertility or MAC was over. Others attempted to limit their excitement, as a form of self-protection: *“I think to a certain extent until he almost turned up, there was part of me that was just like, I guess having gone through the whole IVF thing, I was just like, I just don’t want to get too excited…” *[48]^ (p.25)^.

Doubt and disbelief regarding the pregnancy were expressed [9, 12, 34, 35, 46], linked to previous failed attempts to conceive [27, 35, 51]. Intense states of worry were quickly followed by fears regarding the viability of the pregnancy and miscarriage prominent [11, 12, 27, 34–37, 46, 48, 51], particularly during the first trimester.

The MAC journey influenced reactions to pregnancy: “*It’s taken so long to get here, that it’s not the same, or maybe it is, we don’t know; but a woman who gets pregnant naturally or has no trouble getting pregnant may not think about things as much as someone who’s spent two years going through this process*” [50]^ (p.95)^*.* These reactions led to significant value being placed on the pregnancy [14, 27], with difficulties in imagining a future life with their baby: “*I always feared that something would happen to my child. As if taking it in my arms was impossible…*” [46]^ (p. 4177)^. For some parents, these fears diminished at significant milestones [12, 36], with a growing sense of stability as the pregnancy progressed [35]. However, for others, these worries persisted throughout, switching to fears of stillbirth [34, 37, 51]. In papers that discussed the non-gestating partners’ perspectives (in this case, all male partners’ perspectives) these fears were often less apparent [12, 14, 36], with them feeling reassured by their partners’ changing bodies.

Individuals (i.e., mothers) lacked trust in their own bodies [11, 12] and feared they could not sustain the pregnancy*: “…to be honest I just stopped trusting my body, it never behaves at it should do, if you expect it to do something, it will do something else” *[12]^ (p.573)^*.* These fears often became an obstacle to live the pregnancy fully, impacted bonding: “*Now I am in doubt whether I should bond with her. What if something happens to that or a problem occurs” *[9]^ (p.5)^.

Participants were reluctant to disclose or were eager to minimise the physical and psychological burdens of pregnancy and the impact of MAC treatment [12, 27], due to their long desire to be parents. Some parents viewed pregnancy as a “reward” that “compensated” [27]^ (p.125)^ for the challenges they had encountered. However, studies published in the UK reported a reduced entitlement to complain about the challenges of pregnancy and parenting [12, 48], reflecting potential cultural differences. 

The health of the baby was a dominant theme across studies. Concerns regarding fetal abnormality [11, 34, 35, 37, 51], prematurity [11, 34, 51] and disability [11] were found. These feelings were heightened before or during prenatal visits [35] and often persisted throughout pregnancy [34]. For some, the fear of abnormalities and disabilities disappeared upon greeting their baby [51], whereas for other parents this persisted into the parenting journey, considering their child more vulnerable to illness and injury [43].

#### Subtheme 1.2: Acting to protect

The uncertainty, anxiety and fear associated with pregnancy often led to hypervigilance and monitoring of bodily symptoms [9, 11, 14, 35, 43, 48],with any indication of difficulty destabilising individuals, causing emotional turmoil:

*“I always checked my underwear in bathroom to see if there is any problem. Two weeks ago it seemed that I had some brownish spots. It was the time when I just sat and started crying loudly” *[9]^ (p.5)^. On noticing any physiological changes, individuals swiftly sought the help of health professionals. For expectant fathers, this anxiety was expressed through constant checking and monitoring of their partner: *“Every time (mother) left the room I was like ‘you alright, alright?’ Every time, I was like ‘is everything alright” *[48]^ (p.439)^.

A need for reassurance was evident leading individuals to repeatedly perform pregnancy tests [12, 46, 48, 50] and seek medical help [11, 35]*: “After I learned that I was pregnant, I kept repeating the urine pregnancy tests every day. I was so afraid that the baby was not inside*” [46]^ (p.4177)^. Changes in body shape and fetal movement helped individuals fully realise and believe in their pregnancy [46], increasing their sense of responsibility and feelings of protection [11].

MAC added to the fragility of pregnancy: *“Because he was IVF you kind of think, because he didn’t happen naturally, you’re extra careful and extra cautious because it was like, it is your one shot” *[48]^ (p.439)^. Both parents reported a growing sense of responsibility [9, 14, 36], with the developing baby prioritised: “*I took a LOA for one year and rested in bed for a long time. There was only one focus in my life at the time, the babies inside me. I knew that only I was able to protect the two babies in my belly. I had to guard them with my life*” [35]^ (p.4)^. This responsibility led to acts of protection and modification of lifestyle, such as delaying or reducing physical activity [9, 10, 35, 37, 46], working patterns [11, 35], sexual intercourse [9, 38] and changes to diet [10, 35]. This sense of caution resulted in delayed preparations [11, 12], including arranging the nursery or buying items*: “You know, people keep saying, ‘Have you decorated the nursery?’ and I can’t think of anything worse than coming home to a decorated nursery, you know, if, if things go wrong you know*” [12]^ (p.173)^.

Expectant fathers reported feeling protective of their pregnant partner, “*taking care*” of them [14]^ (p.4)^, by attending appointments, providing emotional and financial support [14]. These feelings of protection persisted into the parental journey [43, 46], with parents attempting to safeguard their infant from harm.

### Theme 2: The stark realisation of the parental dream

The journey to parenthood often starkly contrasted to individuals’ hopes and expectations. For some, the process of becoming a parent was embraced, whereas others struggled to realise their dream. This theme, with its three subthemes, encapsulated individuals’ transition of identity, approaches to parenting and their onward journey, considering future family formation.

#### Subtheme 2.1: Navigating old and new identities

The transition in identity began at the point of conception for some [11]. However, for others this was a complicated process with a “*fragile, obscure and unstable*” identity [35]^ (p.5)^, particularly during the first trimester. Achieving pregnancy did not erase individuals’ conception journey, impacting their ability to embrace their newly emerging identity [10, 34, 37, 46], causing disturbances in the self: *"it may come to something positive [changes brought about by maternity], maybe not, … I lost my personality, my identity…. Who am I?"* [34]^ (p.494)^.

Couples’ views on MAC impacted their ability to accept their new identity expressing concerns about their ability to care for their baby [34]. Some minimised [11, 27, 44] and “*rejected” *[48]^ (p.441)^ their previous infertility and the impact of MAC, whereas others demonstrated acceptance [11, 50].

The birth was a significant moment in the transition of identity. For some, there was a strong desire to birth naturally with feelings of disappointment and regret at requiring medical intervention [51]. Following the birth, feelings of “*shock*” [50]^ (p.70)^ were reported. For many, physically seeing or holding their infant was necessary to realise their parental identity and joy [11, 14] : *"I want to do my best to raise my baby by seeing and touching my baby and realizing that I have become a mother" *[51]^ (p.225)^.

Feeling “*complete*” [35]^ (p.6)^ upon their baby’s arrival and viewing their parenting role as “*amazing*”, or “*incredible*” [48]^ (p.440)^ were reported: “*The joy of having a baby is much greater than winning a lottery... I think that is the greatest happiness in my life. This is something I could never have realised before I became a Mum*” [35]^ (p.6)^. However, the lasting impact of MAC was evident for parents, fading into the background, yet etched in their minds: “*And I remember feeling that I wouldn’t ever forget how hard that actually was to go through the treatment and I don’t think I have to some extent, but I feel like it’s faded a bit into the background” *[48]^ (p.442)^.

Pregnancy and motherhood were considered fundamental to being a woman: *“becoming a mother really meant feeling like a woman” *[46] ^(p.4179)^. Individuals reported feeling more valued because of their pregnancy, increasing self-confidence, reclaiming their sense of self [9, 11, 46] and restoring trust in their bodies’ capability [11, 46].

#### Subtheme 2.2: The need to be a perfect parent, for their precious baby

The pregnancy and baby were often viewed as “*precious*” [48]^ (p.442)^, [46] ^(p.4179)^, “*delicate*” [34]^ (p.118)^, “*special*” [27]^ (p.126)^ or “*highly valued*” [44]^ (p.2)^, impacting approaches to parenting. This presented with parents needing to provide “*perfect*” care [46]^ (p.4179)^, [43] ^(p.48)^, centring their lives around the infant, with parental needs secondary [42, 44, 46]. This parental approach sometimes led to more permissive parenting styles, fulfilling the child’s every wish and a reluctance to provide consequences: *“We would do everything she wanted*” [42]^ (p.1791)^. Not only was the child viewed as highly “special”, but the mother also: *“We are not better mothers, but more special. When you want something so much, you appreciate it and take care of it with more care. I worry a little more about him because he was so longed for” *[40]^ (p.72)^. Gratitude was expressed by parents [48], acknowledging that their experience of MAC changed their “*perspectives*” [48]^ (p.441)^ and tempered the challenges parenthood brings, even helping some parents to maintain a sense of “*calm*” [48]^ (p.441)^ in stressful situations.

The fears of loss apparent in pregnancy overspilt into parenting [42], leading to concern, “*over-protection*” [43] ^(p.48)^ and over-involved parenting [42, 43, 46]. This was characterised by constant monitoring [34, 42, 43] and supervision of their infants: *“I’m very careful, I’m afraid that something bad happens for him, always keep an eye on him and keep him with me” *[42]^ (p.1791)^. Mohammadi et al. (2015) [43] reported an “*over-emotional investment*” characterised by enmeshment of mother and infant*: “He saved my life. Every second I think about him, I’m totally obsessed with his future *[43]^ (p.48)^.

Different approaches to parenthood were noted between male and female partners [44], with fathers referenced as more “*laid back*”, “*playful*” and less protective [44]^ (p.7)^*.* Contrary to this, Allan et al. (2019) [48] reported the unification of parents in their approach to parenthood, with close involvement and negotiation from both parents.

Strong desires to breastfeed were reported [40, 51], with mothers wanting to offer their child the *“best start” *[40]^ (p.70)^. Often individuals endured physical pain and self-sacrifice to continue with their quest to breastfeed*: “The cracks hurt a lot, when I had been breastfeeding for a little, the pain went down a little bit, but I thought about stopping breastfeeding. I made the sacrifice and put up with the pain for him, and I don’t regret it” *[40]^ (p.71)^. Opposingly, prolonged breastfeeding was evident [40, 44], linked to feelings of protection and bonding.

#### Subtheme 2.3: The onward journey

Thoughts and feelings regarding future family formation and the decision to have another child were discussed [42, 46, 48, 50]. Having another child was not viewed as a guarantee [42], with many fearful of re-embarking on the MAC journey [42, 46], reminded of the emotional turmoil [48], pain [46, 48]^ (p.4177)^ and anxiety [46] previously experienced. Attachment to frozen embryos were reported [48] and comments from others regarding a second child were hurtful reminders of their journey to conception [48].

### Theme 3: Psychosocial needs and support

This theme and its two subthemes captured individuals’ psychosocial care needs as they navigated complex health systems, relationships within their own support networks and the impact of sociocultural context.

#### Subtheme 3.1: Unmet care needs

Whilst the desire to receive routine antenatal care [30] and to be treated *“like everyone else” *[12]^ (p.574)^ were presented, the care needs of some individuals contradicted this [12, 30]. Discharge from the fertility centre brought satisfaction, signalling the end of their MAC journey [30]. However, ambivalence regarding the transition was evident, with “*gaps in care*” reported [12]^ (p.574)^. This was often an anxious time for individuals lacking in containment: *“We didn’t really know who to turn to, we hadn’t seen our midwife yet and we needed to know everything was okay with the pregnancy, so we paid for a private dating scan*” [12]^ (p.574)^.

Participants’ care needs were not adequately addressed by routine services, with little understanding or attention from health professionals regarding their conception journey [12, 30]. Increased care needs [30] were reported by individuals requiring additional appointments and scans to contain their anxiety. There were barriers to discussing their concerns with health professionals in fear of being perceived as “*ungrateful*” [12]^ (p.105)^; parents reported often requiring prompts from professionals to initiate discussions [30]. Nevertheless, certain qualities were valued in health professionals, such as knowledge regarding their conception journey, personalised care, clear and consistent communication, more frequent appointments, understanding and reassurance [10, 12, 30].

#### Subtheme 3.2: The journey from exclusion and marginalisation to acceptance and belonging

Feelings of difference were experienced in pregnancy after MAC [9, 11, 12, 46, 48]. “*Resentment*” [48]^ (p.441)^ towards spontaneously conceiving couples was expressed, feeling others could not understand their journey to conception [12, 46], or the emotions that pregnancy brings [46]. The uncertainty associated with pregnancy often translated into reluctance to share their pregnancy news with others [9, 11, 35, 36, 50]: “*only tell immediate family and friends and then if it hasn’t worked, we can then be open and honest again, once we’ve kind of healed a bit” *[10]^ (p.370)^.

Achieving pregnancy brought complex emotional reactions and a sense of betrayal to their previous childless identity [9, 12]. Reports of guilt, empathy and sadness for those still trying to achieve pregnancy were evident [9, 11]. Parents also reported “*finding meaning in life*” [9]^ (p.4)^ once pregnancy was achieved, shifting from a position of isolation to connection, feeling supported by others such as health professionals, family and friends [11, 46], bringing hope and optimism [9].

Going through MAC and achieving pregnancy was reported to strengthen marital relationships [9, 37, 38, 50], with the partner reported as the biggest source of support during pregnancy[35]. A reduction in conflict, improved communication and understanding were expressed by individuals [38]. Furthermore, having a biological child led to greater feelings of acceptance and connection with the partner’s family: “*I was finally connected with my husband’s family after having the baby, and the baby represented a biological link. Otherwise, I would have always been an outsider” *[35]^ (p.6)^.

As the pregnancy progressed, individuals relied on family and close friends [48] to provide practical and emotional support, both antenatally and postnatally [9, 11, 35, 37, 40]. Furthermore, making new connections with other parents who had conceived via MAC was a welcomed bonding experience [48] facilitated by internet forums [10].

Sociocultural norms regarding conception influenced individuals’ ability to integrate their former and current selves [48]. Having a child seemed to hold cultural significance, with the impact of society particularly evident in papers conducted in Asia. In some instances, individuals never disclosed their use of MAC in achieving pregnancy due to fears of shame and negativity from their community [9, 42]. Individuals felt free from stigmatisation on achieving pregnancy [9], feeling more accepted by others and society, increasing self-worth: *“In every party or religious ceremony people came to me and said we bring this for you because of your pregnancy. The feeling and opinion of people toward me has changed now*” [9]^ (p.4)^.

The role of religion and spirituality was reflected upon [9, 35, 42, 43], helping individuals manage and cope with the uncertainty and doubt that accompanied pregnancy: *“Sometimes I feel that I can do nothing more myself. I read holy Quran and say prayers. I rely on the strength from God” *[9]^ (p.6)^. Parents prayed for a successful pregnancy [9, 35] and, in some instances, attributed the birth to God [43].

## Discussion

This study achieved its aim of exploring the experiences of pregnancy and early parenting in individuals who conceived via MAC, identifying their psychological, social and health care needs. We were unable to identify another review that qualitatively synthesised the antenatal and parenting experiences of both parents who conceive via MAC; therefore, to the researchers’ knowledge, this is the first review of its kind. Overall, achieving pregnancy brought complex emotional reactions and lacked in a sense of safety, with amplified care needs identified by parents. The health and survival of the baby preoccupied parents’ thoughts, leading to acts of protection and cautious living, impacting their transition to parenthood and their identity as a parent. This transition to parenthood can be a lengthy, non-linear process, influenced by health systems and the sociocultural context.

This review confirmed that pregnancy following MAC was associated with pregnancy-specific anxiety, fears of pregnancy loss and concerns regarding the health of the baby [5, 7, 8]. This heightened state of threat impacts parents’ ability to imagine a life with their baby and, in some cases, impacts bonding. This is significant, given maternal representations during pregnancy are predictors of attachment one year postnatally [53]. Furthermore, pregnancy is a critical time for the neurodevelopment of the developing baby. According to the ‘fetal origins hypothesis’ the utero environment can have a sustained impact across the lifespan [54]. Prenatal maternal anxiety can impact the neurobehavioural development of the fetus [55–57], with long-term effects observed on the child’s cognitive ability [57, 58] and emotional adjustment [59]. The findings from this review may have implications for clinical guidance, suggesting a need for early identification and monitoring of psychological distress, with targeted interventions to reduce pregnancy-specific anxiety. These findings hint at the potential value in identifying pregnancy following MAC as a risk factor for emotional vulnerability, which may need to be considered for any future revisions to antenatal and postnatal guidance. However, more studies in this area are required to further support and substantiate this recommendation.

Perceived social stigma impacted parents’ relationship to help. Hammarberg et al.’s mixed methods review [5] suggested individuals might “idealise” [5]^(p.411)^ parenthood with a reduced sense of entitlement to complain about the negative aspects of parenting. The current review identified this notion both antenatally and postnatally. French et al. [12] referred to this as “self-silencing” [12]^ (p.574)^. Self-silencing or the internalisation of difficulties has been linked to depression [60–62], lower levels of self-esteem and disturbances in identity [63]. This finding is important given the potential negative consequences for maternal mental health and the mother-baby relationship [64]. Normalising parental experiences and reducing stigma and shame regarding MAC are important milestones for health services and society to achieve.

The transition to parenthood was often complex for expectant parents, facing unique challenges. Maehara et al. (2021) [8] made similar observations in their review, suggesting the acquisition of the maternal identity was delayed, defending against any anticipated disappointments and loss. Individual variability regarding the transition of identity was noted across studies. For some parents, the transition in identity began at the point of conception [11], whereas others struggled to realign their old and new identities, needing to physically see and hold their infant to realise their dream of having a baby. Shifts in identity have been linked to anxiety and confusion [65], making this an important issue to identify, assess and support individuals with.

This review identified the significant role of culture and society in shaping experiences of pregnancy and parenting following MAC. Individual, familial and cultural values, regarding reproduction and gender, impact acceptance of MAC and the transition of identity. The sociocultural context featured heavily in papers written in countries with collectivist cultures, amid societal pressures regarding child-rearing [35, 42, 43]. Parents felt ostracised by their previous childless identity and stigmatised for their use of MAC, impacting help-seeking from services and from their own support networks. Greater acknowledgement of the sociocultural factors impacting expectant parents are necessary within health services.

This meta-synthesis was novel because it qualitatively synthesised the experiences of both parents following MAC. Four of the included papers reviewed the experiences of couples and two focused specifically on the male partner’s experiences. The reviewed literature suggested fears of pregnancy loss were less apparent in male partners. However, there was indication of anxiety and hypervigilance in fathers which was expressed through checking and monitoring the health status of their partner [48]. A sense of growing responsibility was reported for both parents, with male partners expressing this via acts of protection towards the gestating partner antenatally. This finding suggests a potential need for health services to monitor the emotional well-being of both parents following MAC. Our search identified two studies [14, 44] which focused specifically on the father’s perspective. The authors [14, 44] suggested that there were possible differences in the approaches to parenting across parental couples, with the fathers self-identifying as more relaxed and ‘playful’. However, given the limited number of studies in this field, more research is required on possible parental differences.

This meta-synthesis enhanced and extended the findings of Maehara et al. [8], identifying that individuals’ conception journey persisted into parenthood, leading to acts of protection. These acts of protection may be individuals’ attempts to reclaim an internal locus of control [66] to combat the uncertainty and loss of control during MAC and pregnancy. Quantitative studies have reported varied responses in adjustments to parenting [5], with a suggestion of no differences compared to spontaneously conceiving mothers [67]. However, the reviewed qualitative literature suggests that mothers who conceived via MAC experience a need to provide idealised and “*perfect care*” [43, 46] with high levels of psychological investment in their child. According to Meighan’s Becoming a Mother Theory [68], parents or mothers mostly are required to establish responsibilities and boundaries for themselves and their baby. Failure to integrate and process their conception journey may impact this process, leading to over-involved, over-protective, permissive parenting styles with the potential for enmeshment [69] and co-dependency [70], affecting attachment [71], the infant’s sense of autonomy [69], potentially hindering their social and emotional development [72–74]. Furthermore, it highlighted that parents’ experiences of MAC impacted decisions regarding future family formation.

### Strengths and limitations

The meta-synthesis approach allows for new interpretation of multiple findings, which can be used to inform clinical practice. Scientific rigour and transparency were core features of this review with efforts made to reduce bias at screening, data extraction and quality appraisal, with the incorporation of independent reviewers. This review included studies written in different languages, allowing the incorporation of experiences of individuals from different countries and cultures. Similarities were identified across the included studies, suggesting an acceptance and universality of experiences internationally. The findings explored a broad range of experiences both during pregnancy and parenting, something that has not been done before.

Whilst this meta-synthesis focused on the experiences of pregnancy and parenting following MAC, some caution is necessary for the interpretations and generalisation of previous findings to this study. The review identified a bias of MAC in the context of subfertility with 19 of the included papers recruiting only participants with a history of fertility challenges. Therefore, it is difficult to disentangle whether their experiences are the result of MAC, subfertility, or an interaction of both.

Grey literature was excluded in the belief that its methodological quality could be weaker. However, we acknowledge that this decision might have introduced publication bias. The exclusion of grey literature and mixed methods studies could have resulted in the possibility of some relevant papers being missed. Although the majority of studies had good methodological quality, thereby enhancing the trustworthiness of findings, none of the included studies sufficiently referred to reflexivity, an essential component in qualitative literature. Only two studies [37, 51] received a low quality rating; however, the knowledge extracted from these papers did not heavily influence the overall findings. Despite best efforts to include all relevant results, three studies met all other relevant inclusion criteria but failed to report the method of MAC [75–77]. Thus, it was not possible to ensure that the study sample included individuals pregnant/parenting after advanced MAC techniques (as opposed to after OI/IUI only). Attempts to clarify this information with the authors were unsuccessful. It is possible these studies were relevant to the review but were excluded on this basis. Finally, six articles (two Japanese, four Portuguese) were translated into English. Whilst these were checked for accuracy by native speakers, these were not professional transcribers or translators; therefore, meaning may have been lost in translation.

There was a lack of consistency across papers in reporting the ethnicity of participants. Additionally, when ethnicity was reported, most participants were from white backgrounds. Therefore, the views of parents from black and minority ethnic (BAME) groups remain under-represented in the literature.

### Research implications

Whilst the findings identify the challenges and difficulties experienced by groups of parents, further research is necessary to determine what kind of support individuals find helpful during pregnancy and parenting. As this review identified a significant bias towards MAC in the context of infertility, further research is necessary with mixed samples or those who sought MAC for alternate reasons, such as being in a same-sex couple or a solo parent. Qualitatively exploring the perspectives of these individuals would allow a more holistic picture, further disentangling the impact of MAC and infertility on experience. Furthermore, the views and experiences of parents from BAME backgrounds are under-represented in the current literature, which is a priority for future research.

### Clinical implications

As part of this systematic review, the psychological, social and health care needs of individuals during pregnancy and parenting following MAC were extracted and synthesised, with suggested clinical recommendations (Table 4).

**Table 4:** Psychological, social and health care needs of parents following MAC with clinical recommendations

	**Needs**	**Clinical recommendations**
Psychological	Potential anxiety in pregnancy	• Increased awareness of potential psychological distress and adjustment difficulties amongst health professionals in maternity services who provide care for individuals pregnant after MAC • Psychological service provision which validates the potential psychological challenges within this client group • Clinical Health Psychologists embedded within maternity services to disseminate psychological thinking, provide containment and intervention on an individual and systemic level • Training for health professionals in identifying, monitoring and screening of emotional well-being for all individuals who successfully achieve pregnancy following MAC • The offer of psychological support during MAC and resultant pregnancy to help individuals integrate and process their experiences, if required • Consideration of the longer-term psychological needs to be considered incorporating difficulties relating to transition in identity and role. This could be achieved via psychologically informed antenatal and parenting groups or the offer of one-to-one therapeutic sessions focusing on the parent-infant relationship and bond
Social	The possibility of feeling excluded and marginalised Possible reluctance to share distress and experiences due to fear of judgement, shame and stigma	• Normalisation and peer support groups to be offered to help reduce feelings of exclusion • Incorporation of sociocultural factors such as religious values and beliefs when conducting assessment • Individuals to be signposted to appropriate support services, if required
Healthcare	Reassurance and containment	• Promotion of consistency of care with MAC-aware midwives (in the absence of indication for consultant led care) antenatally and postnatally where individuals can develop trusting relationships with familiar health professionals • Specialist training provided to midwives and consultants in relation to pregnancy and parenting after medicalised conception and infertility • Health professionals to be proactive in fostering non-judgemental spaces, enquiring about the impact of MAC, validating experiences

## Conclusions

This review highlights the significant psychosocial impact of MAC impacting antenatal and early parenting experiences, exacerbated by the sociocultural context. As evidenced, this can lead to unmet care needs, delayed help-seeking and acts of protection. A need for increased monitoring of parents’ emotional well-being and adjustment to pregnancy and parenting is required. Continuity of care and specialist training for health professionals is necessary to ensure services identify and meet the needs of those who successfully conceive via MAC.

## Supplementary Information

**Additional file 1: Appendix 1. **The ENTREQ Checklist (Tong et al., 2012)

**Additional file 2: Appendix 2. **Search strategy for each database

**Additional file 3: Appendix 3. **Matrix of themes

## Data Availability

The datasets generated and/or analysed during this study are not publicly available due to lack of consent for participants to make whole interview transcripts available. But upon reasonable request they can be made available from the first and/or corresponding author.

## References

[1] Human Fertilisation and Embryology Authority (2019, May). Fertility treatment 2017: trends and figures. https://www.hfea.gov.uk/media/2894/fertility-treatment-2017-trends-and-figures-may-2019.pdf .

[2] Centre for Disease Control and Prevention. (2021). *Assisted Reproductive Technology: Fertility Clinic and National Summary Report.* Accessed 11/12/2021: https://www.cdc.gov/art/reports/2019/pdf/2019-Report-ART-Fertility-Clinic-National-Summary-h.pdf.

[3] Wyns C, De Geyter C, Calhaz-Jorge C, Kupka MS, Motrenko T, Smeenk J, Goossens V (2021). ART in Europe, 2017: results generated from European registries by ESHRE. Human Reproduction Open.

[4] National Perinatal Epidemiology and Statistics Unit. (2021, September). *Assisted reproductive technology in Australia and New Zealand.* The University of New South Wales. https://npesu.unsw.edu.au/sites/default/files/npesu/data_collection/Assisted%20Reproductive%20Technology%20in%20Australia%20and%20New%20Zealand%202019.pdf.

[5] Hammarberg K, Fisher JRW, Wynter KH (2008). Psychological and social aspects of pregnancy, childbirth and early parenting after assisted conception: a systematic review. Hum Reprod Update.

[6] Hjelmstedt A, Widström A, Wramsby H, Collins A (2003). Patterns of emotional responses to pregnancy, experience of pregnancy and attitudes to parenthood among IVF couples: A longitudinal study. J Psychosom Obstet Gynecol.

[7] Gourounti K (2016). Psychological stress and adjustment in pregnancy following assisted reproductive technology and spontaneous conception: A systematic review. Women Health.

[8] Maehara K, Iwata H, Kimura K, Mori E. Experiences of transition to motherhood among pregnant women following assisted reproductive technology: a qualitative systematic review. Joanna Briggs Institute of Evidence Synthesis. 2021. p. 1–39*. *10.11124/jbies-20-00545. 10.11124/JBIES-20-00545 34410230

[9] Ranjbar F, Akhondi MM, Borimnejad L, Ghaffari SR, Behboodi-Moghadam Z (2015). Paradox of modern pregnancy: a phenomenological study of women’s lived experiences from assisted pregnancy. J Pregnancy.

[10] Walker C, Mills H, Gilchrist A (2017). Experiences of physical activity during pregnancy resulting from in vitro fertilisation: An interpretative phenomenological analysis. J Reprod Infant Psychol.

[11] Hayashi H, Sayama M. Emotional processes during pregnancy among women successfully conceived via assisted reproductive technology. J Jpn Society of Midwifery. 2009;23(1):83–92.

[12] French LR, Sharp DJ, Turner KM (2015). Antenatal needs of couples following fertility treatment: a qualitative study in primary care. Br J Gen Pract.

[13] National Institute for Health and Care Excellence. (2017). *Fertility problems: assessment and treatment* [NICE Guideline No. CG156]. https://www.nice.org.uk/guidance/cg156. 32134604

[14] Sonego JC, Dornelles LMN, Lopes RDCS, Piccinini CA, Passos EP. A experiência paterna da gestação no contexto da reprodução assistida. Psicologia: Teoria e Pesquisa; 2017;32(4):1–9.

[15] Moher, D., Liberati, A., Tetzlaff, J., Altman, D. G., & PRISMA Group (2009). Preferred reporting items for systematic reviews and meta-analyses: the PRISMA statement. Ann Intern Med.

[16] Page M, McKenzie J, Bossuyt P, Boutron I, Hoffmann T, Mulrow C, Shamseer L, Tetzlaff J, Akl E, Brennan S, Chou R (2021). The PRISMA 2020 statement: an updated guideline for reporting systematic reviews. Int J Surg.

[17] Tong A, Flemming K, McInnes E, Oliver S, Craig J (2012). Enhancing transparency in reporting the synthesis of qualitative research: ENTREQ. BMC Med Res Methodol.

[18] Cooke A, Smith D, Booth A (2012). Beyond PICO: the SPIDER tool for qualitative evidence synthesis. Qual Health Res.

[19] Critical Appraisal Skills Programme. 2018. CASP Qualitative Checklist. [online]. Available at: https://casp-uk.net/wp-content/uploads/2018/01/CASP-Qualitative-Checklist-2018.pdf. Accessed Sept 2021.

[20] Long HA, French DP, Brooks JM (2020). Optimising the value of the critical appraisal skills programme (CASP) tool for quality appraisal in qualitative evidence synthesis. Research Methods in Medicine & Health Sciences.

[21] Butler J, Gregg L, Calam R, Wittkowski A (2020). Parents' Perceptions and Experiences of Parenting Programmes: A Systematic Review and Metasynthesis of the Qualitative Literature. Clin Child Fam Psychol Rev.

[22] Thomas J, Harden A (2008). Methods for the thematic synthesis of qualitative research in systematic reviews. BMC Med Res Methodol.

[23] Thorne S, Jensen L, Kearney MH, Noblit G, Sandelowski M (2004). Qualitative metasynthesis: Reflections on methodological orientation and ideological Agenda. Qual Health Res.

[24] Fletcher AJ. Applying critical realism in qualitative research: Methodology meets method. Int J Soc Res Methodol, 2017;20(2):181–94. 10.1080/13645579.2016.1144401.

[25] Creswell JW (2009). Research design: Qualitative, quantitative and mixed methods approach.

[26] Dodgson JE (2019). Reflexivity in qualitative research. J Hum Lact.

[27] Dornelles LMM, F.: Lopes, R. C.: Piccinini, C. A.: Passos, E. P. (2016). The experience of pregnancy resulting from Assisted Reproductive Technology (ART) treatment: A qualitative Brazilian study. Women & Birth: Journal of the Australian College of Midwives.

[28] Laville C, Dionne J. The construction of knowledge: manual of research methodology in human sciences. trans. Heloísa Monteiro e Francisco Settineri. Porto Alegre: Artmed; 1999.

[29] Smith F, P., & Larkin, M. (2009). Interpretative phenomenological analysis: Theory, method and research.

[30] Warmelink JC, Adema W, Pranger A, De Cock TP (2016). Client perspectives of midwifery care in the transition from subfertility to parenthood: a qualitative study in the Netherlands. J Psychosom Obstet Gynecol.

[31] Boeije H. Analyzing in qualitative research. Thinking and doing. Amsterdam: Boom; 2005.

[32] Patton MQ. Qualitative evaluation and research methods. Newbury Park: Sage Publications; 1990.

[33] Van, M. M. (1990). Van Manen, Max, Researching Lived Experience: Human Science for an Action Sensitive Pedagogy. Albany: State University of New York Press (1990). with new Preface.

[34] Dornelles LMM, F.: Sobreira Lopes, R. C.: Piccinini, C. A.: Passos, E. P. (2014). "Living each week as unique": Maternal fears in assisted reproductive technology pregnancies. Hum Reprod.

[35] Lin YN, Tsai YC, Lai PH (2013). The experience of Taiwanese women achieving post-infertility pregnancy through assisted reproductive treatment. Fam J.

[36] Smorti M, Smorti A (2013). Medical successes and couples’ psychological problems in assisted reproduction treatment: a narrative based medicine approach. J Matern Fetal Neonatal Med.

[37] Dornelles LMN, Lopes RDCS (2011). Will I manage to go through this pregnancy to full term? The maternal experience during pregnancy in the context of assisted reproduction. Estudos de Psicologia (Campinas).

[38] Silva IM, Lopes RDCS. Marital relationship in the context of assisted reproduction: treatment and pregnancy. Psicologia: Teoria e Pesquisa; 2011;27(4):449–57. 10.1590/S0102-37722011000400008.

[39] Colaizzi PF. Psychological research as the phenomenologist views it. Ronald S. Valle, Mark King, eds. Existential Phenomenological Alternatives for Psychology: Exploring the breadth of human experience 1989. Boston: Springer US; 1978. p. 3-16.

[40] DíazSáez J, Fernández-Medina IM, Granero-Molina J, Fernández-Sola C, Hernández-Padilla JM, López-Rodríguez MM (2021). Breastfeeding Experiences in First-Time Mothers After Assisted Conception. Breastfeed Med.

[41] Gadamer HG. Verdad y método [True and Method]*.* Salamanca: Sígueme; 2005.

[42] Sadeghi T, Mohammadi N, Mohammadpour A, Abbasi M (2019). Over-challenging Mothers: Motherhood after Assisted Reproductive Technology. Journal of Midwifery and Reproductive Health.

[43] Mohammadi N, Shamshiri M, Mohammadpour A, Vehviläinen-Julkunen K, Abbasi M, Sadeghi T (2015). ‘Super-mothers’: the meaning of mothering after assisted reproductive technology. J Reprod Infant Psychol.

[44] Bracks-Zalloua P, McMahon C, Gibson F (2011). IVF-Conceiving Fathers' Experiences of Early Parenthood. J Relationships Res.

[45] Boggadan RC, Biklen SK (1998). Qualitative research in education.

[46] Boz İ, Teskereci G, Akgün M (2021). The experience of becoming a mother following successful in vitro fertilization: A grounded theory. J Adv Nurs.

[47] Charmaz K. Constructing grounded theory, 2nd ed. London: SAGE Publications; 2014.

[48] Allan H, Mounce G, Culley L, van den Akker O, Hudson R (2019). Transition to parenthood after successful non-donor in vitro fertilisation: The effects of infertility and in vitro fertilisation on previously infertile couples’ experiences of early parenthood. Health.

[49] Frost N (2010). Qualitative Research Methods in Psychology: Combining Core Approaches.

[50] Crespo E, Bestard J (2016). Psychosocial needs of women and their partners after successful assisted reproduction treatment in Barcelona. Reproductive Biomedicine & Society Online.

[51] Katsumara Y, Kamiya S, Emisu F (2014). Experiences of women who became pregnant with fertility treatment: From pregnancy to puerperium and childcare for a first child. Nihon Josan Gakkaishi.

[52] Greg M, Asahara K, Yokoyama M (2007). How to proceed and summarize qualitative research Aiming to be an expert in nursing research.

[53] Fonagy P, Steele H, Steele M (1991). Maternal representations of attachment during pregnancy predict the organization of infant-mother attachment at one year of age. Child Dev.

[54] Barker DJP (1995). Fetal origins of coronary heart dis-ease. BMJ.

[55] Monk C, Fifer WP, Myers MM, Sloan RP, Trien L, Hurtado A (2000). Maternal stress responses and anxiety during pregnancy: effects on fetal heart rate. Dev Psychobiol.

[56] Van den Bergh BR, Marcoen A (2004). High antenatal maternal anxiety is related to ADHD symptoms, externalizing problems, and anxiety in 8-and 9-year-olds. Child Dev.

[57] Kinsella MT, Monk C (2009). Impact of maternal stress, depression & anxiety on fetal neurobehavioral development. Clin Obstet Gynecol.

[58] Talge, N. M., Neal, C., Glover, V., & Early Stress, Translational Research and Prevention Science Network: Fetal and Neonatal Experience on Child and Adolescent Mental Health (2007). Antenatal maternal stress and long-term effects on child neurodevelopment: how and why?. J Child Psychol Psychiatry.

[59] O'Connor, T. G., Heron, J., Golding, J., Glover, V., & AL Spac Study Team (2003). Maternal antenatal anxiety and behavioural/emotional problems in children: a test of a programming hypothesis. J Child Psychol Psychiatry.

[60] Cramer KM, Thomas N (2003). Factor structure of the silencing the self-scale in women and men. Personality Individ Differ.

[61] Whiffen VE, Foot ML, Thompson JM (2007). Self-silencing mediates the link between marital conflict and depression. J Soc Pers Relat.

[62] Maji S, Dixit S (2019). Self-silencing and women’s health: A review. Int J Soc Psychiatry.

[63] Jack DC, Dill D (1992). The Silencing the Self Scale: Schemas of intimacy associated with depression in women. Psychol Women Q.

[64] Alhusen J, Haya, t M., Gross, D. (2013). A longitudinal study of maternal attachment and infant developmental outcomes. Arch Women’s Mental Health.

[65] Olshansky EF (2003). A theoretical explanation for previously infertile mothers’ vulnerability to depression. J Nurs Scholarsh.

[66] Rotter JB (1954). Social learning and clinical psychology.

[67] Repokari L, Punamäki R-L, Poikkeus P, Tiitinen A., Vilska S, Unkila-Kallio L, Tulppala M. Ante- and perinatal factors and child characteristics predicting parenting experience among formerly infertile couples during the child’s first year: A controlled study. J Family Psychol. 2006;20:670–9. 10.1037/0893-3200.20.4.670. 10.1037/0893-3200.20.4.670 17176203

[68] Meighan M. Maternal role attainment- Becoming a mother. In M. R. Alligood (Ed.), Nursing theorists and their work (9th ed). Elsevier - Health Sciences Division; 2017. p 432-44.

[69] Minuchin S (1974). Families & Family therapy.

[70] Beattie M. Codependent no more: How to stop controlling others and start caring for yourself. Hazelden Publishing; 1992.

[71] Bowlby J (1961). Processes of mourning. Int J Psychoanal.

[72] Wells M, Glickauf-Hughes C, Jones R (1999). Codependency: A grass roots construct's relationship to shame-proneness, low self-esteem, and childhood parentification. Am J Fam Ther.

[73] Sack JL (2000). The Impact of Codependency on Relationships. Theologia Diakonia.

[74] Colpin H, Soenen S (2002). Parenting and psychosocial development of IVF children: a follow-up study. Hum Reprod.

[75] Huang MZ, Sun YC, Gau ML, Puthussery S, Kao CH (2019). First-time mothers’ experiences of pregnancy and birth following assisted reproductive technology treatment in Taiwan. J Health Popul Nutr.

[76] Asante-Afari, K., Doku, D.T. and Darteh, E.K., 2022. Transition to motherhood following the use of assisted reproductive technologies: Experiences of women in Ghana. Plos one, 17(4)*,* p.e0266721. Doi.org/10.1371/journal.pone.0266721. 10.1371/journal.pone.0266721 PMC9032403 35452460

[77] Barnes M (2013). Experiences of birth and breastfeeding following assisted conception. Breastfeed Rev.

