# Supplementary figures and images for: Parents’ experiences of life after medicalised conception: a thematic meta-synthesis of the qualitative literature
Source: BMC Pregnancy Childbirth. 2023 Jul 17;23:520. doi: 10.1186/s12884-023-05727-x (PMC10351127; doi:10.1186/s12884-023-05727-x)

***Appendix 2: Search strategy for each database***


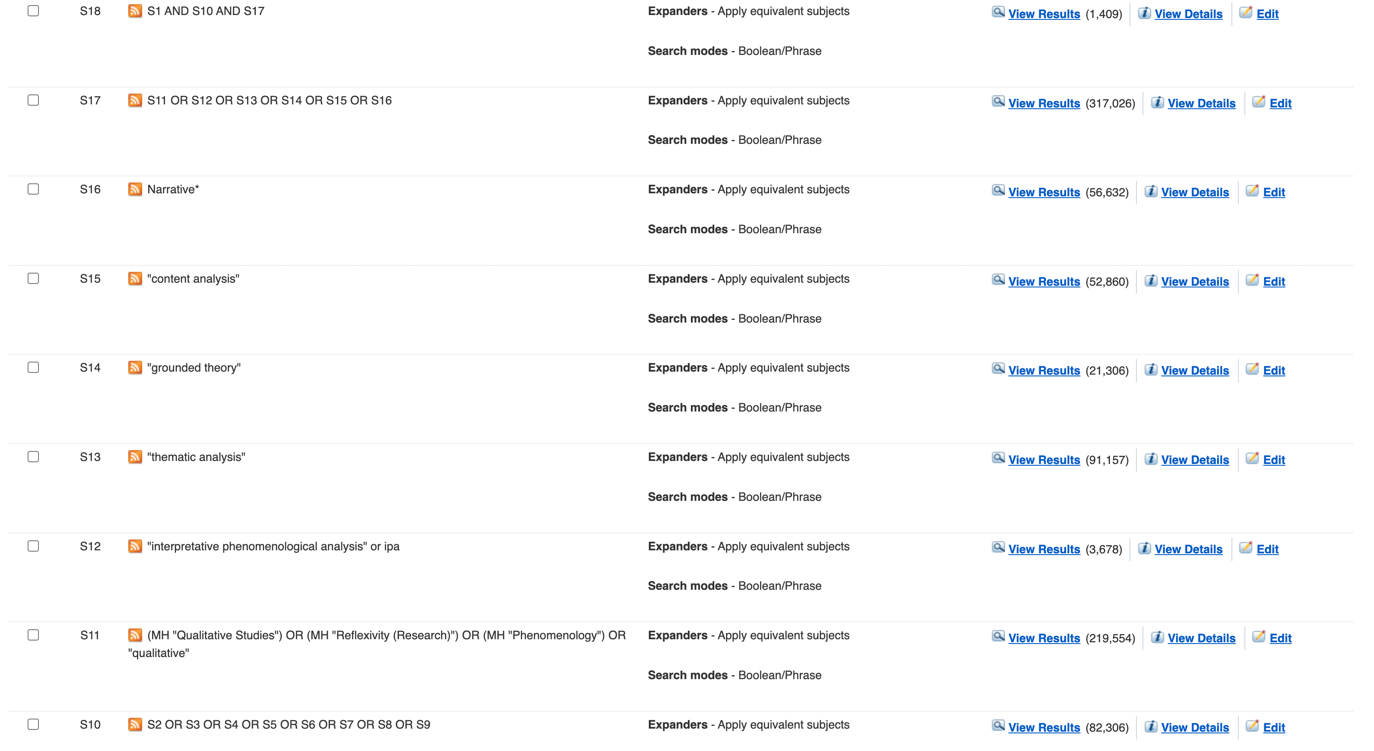
*CINAHL*


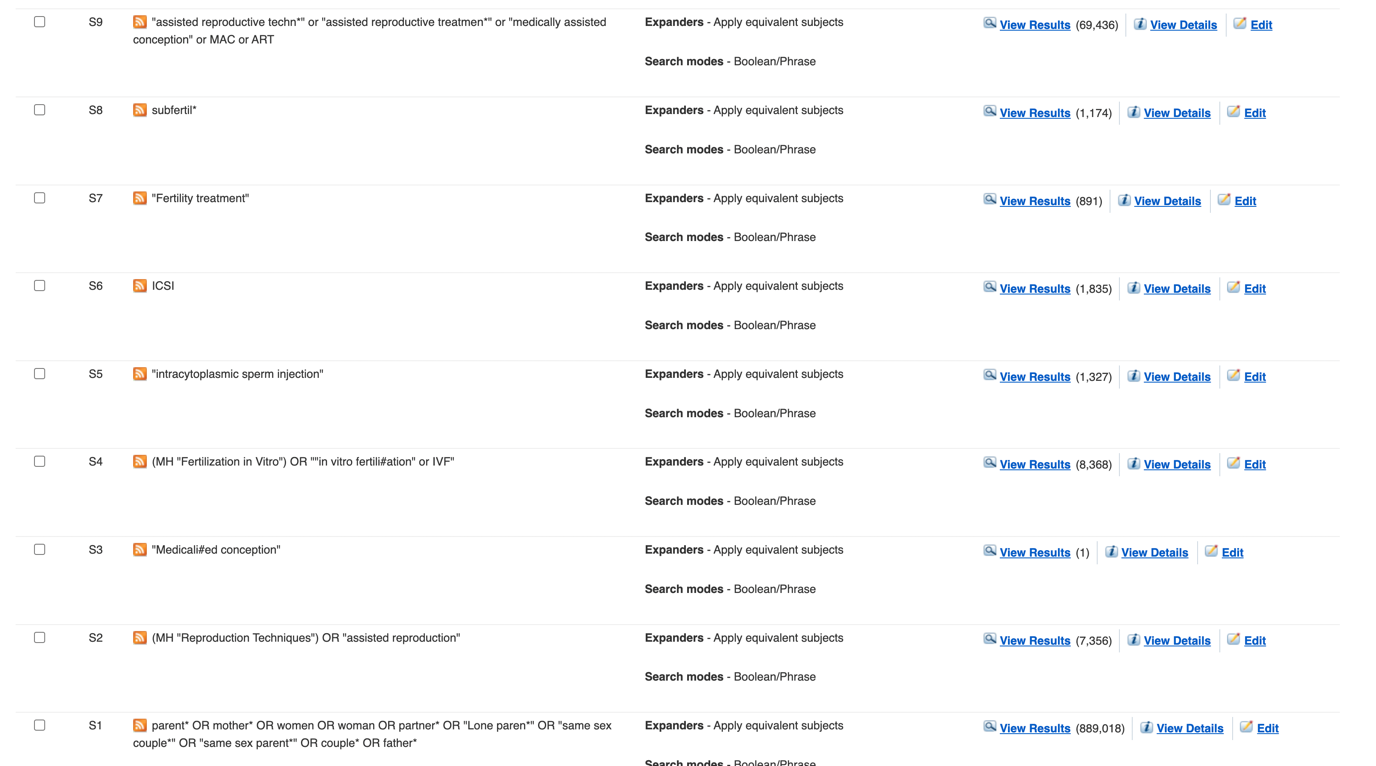


*Medline
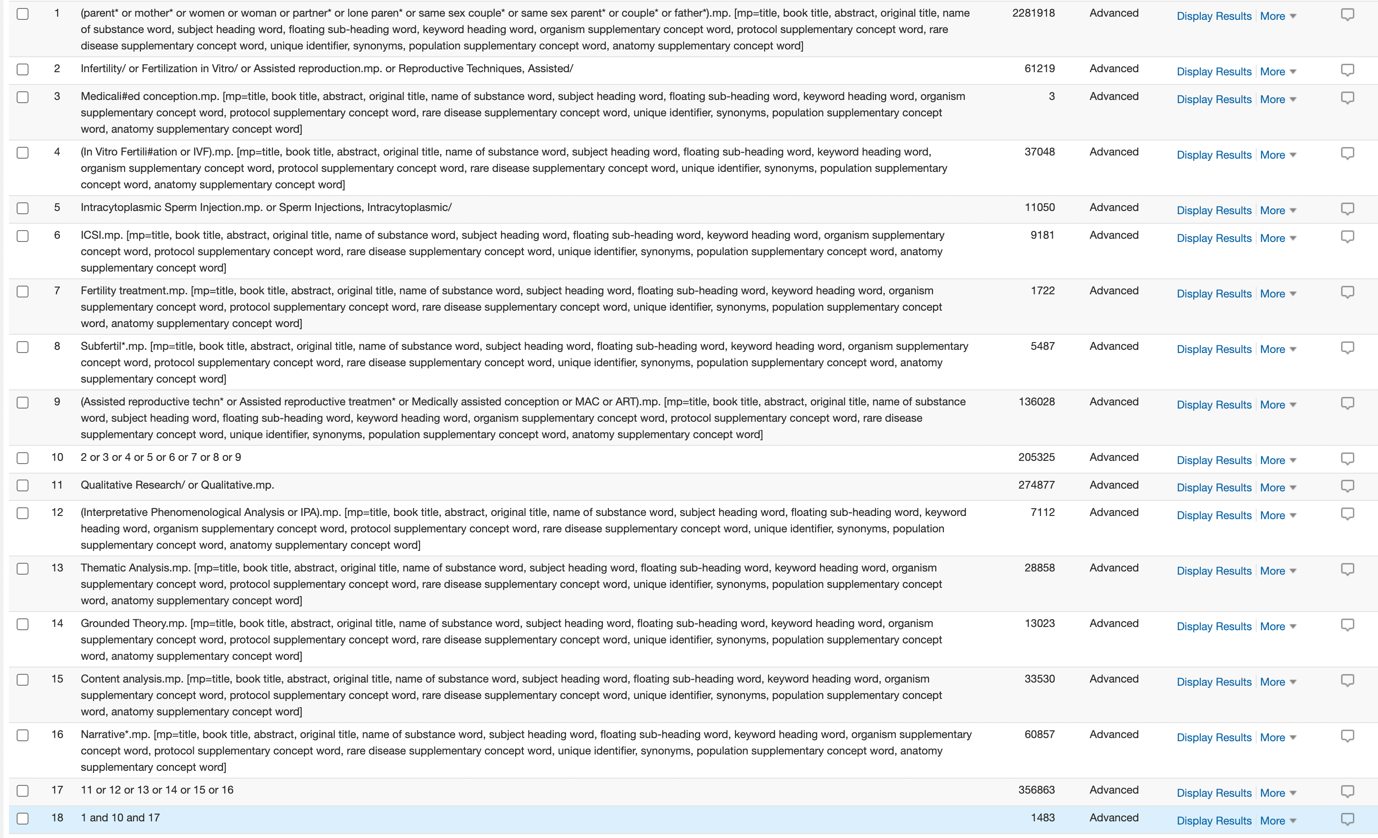
*

*
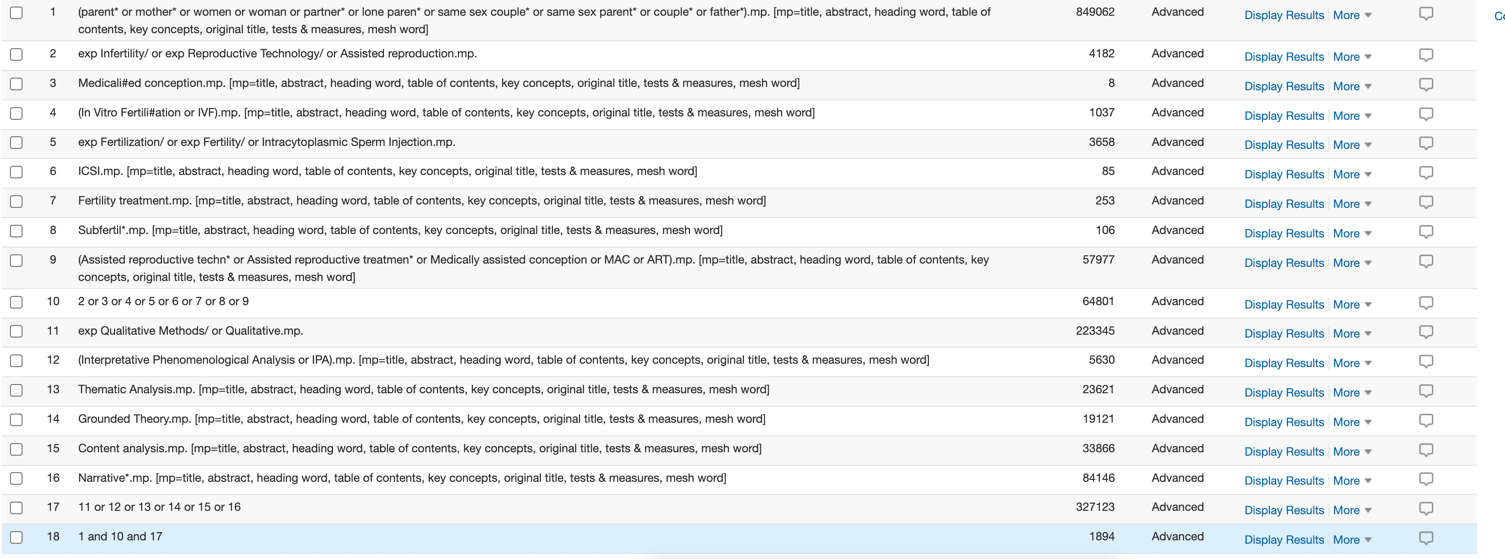
PsycINFO*

*EMBASE*

*
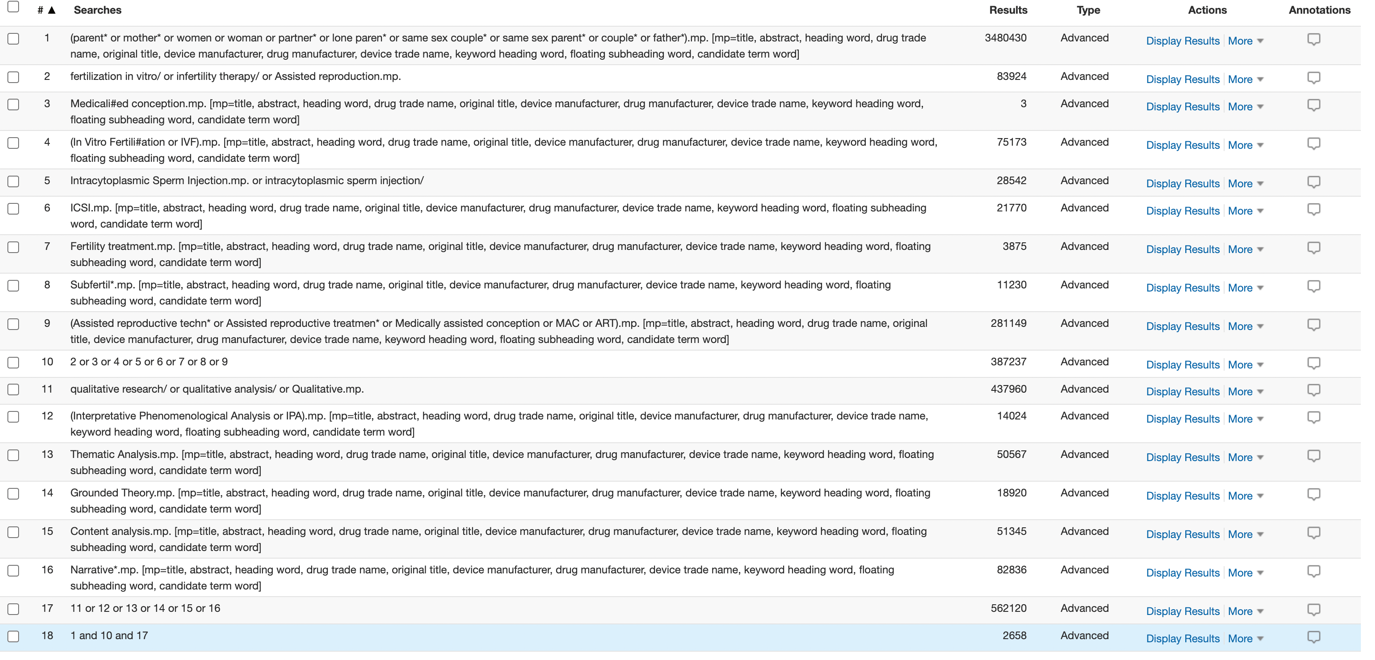
*

*
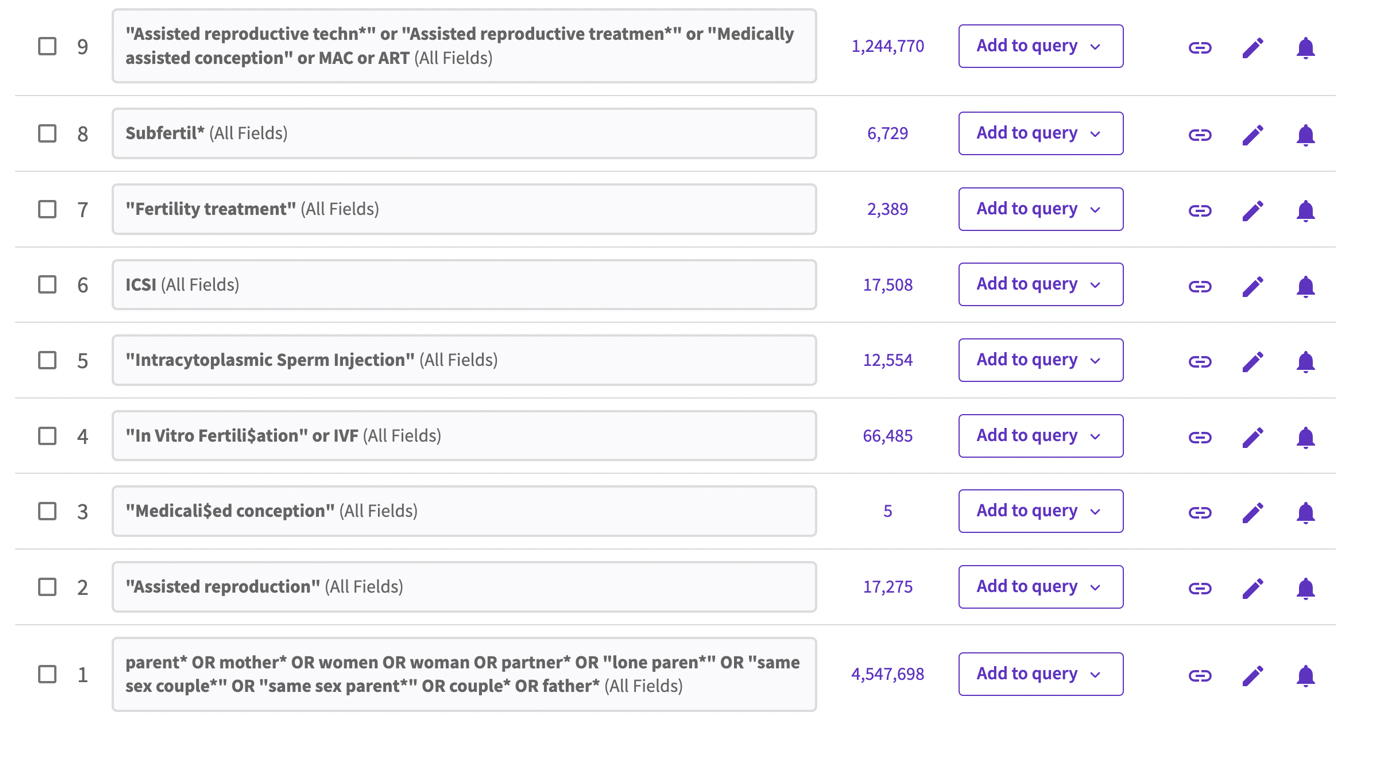

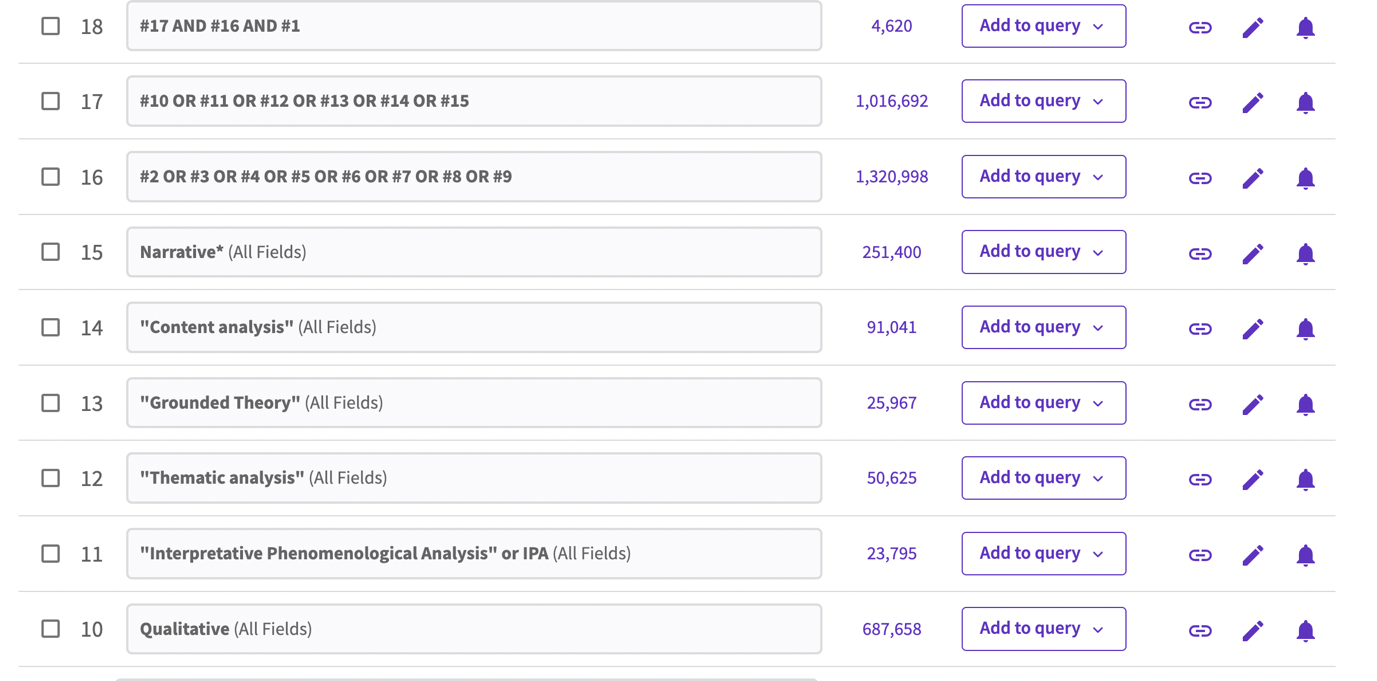
Web of Science*

Supplement: Supplementary file 2 — Additional file 2: Appendix 2. Search strategy for each database [file 12884_2023_5727_MOESM2_ESM.docx]
