# Supplementary material for: Parents’ experiences of life after medicalised conception: a thematic meta-synthesis of the qualitative literature
Source: BMC Pregnancy Childbirth. 2023 Jul 17;23:520. doi: 10.1186/s12884-023-05727-x (PMC10351127; doi:10.1186/s12884-023-05727-x)
Supplement: Supplementary file 3 — Additional file 3: Appendix 3. Matrix of themes [file 12884_2023_5727_MOESM3_ESM.docx]

Appendix 3: Matrix of themes

|  | **Theme 1: the vulnerable Parent** | | **Theme 2: The Stark Realisation of the Parental Dream** | | | **Theme 3: Psychosocial needs and Support** | |
| --- | --- | --- | --- | --- | --- | --- | --- |
|  | Lacking in a sense of safety | Acting to protect | Navigating old and new identities | The need to be a perfect parent, for their precious child | The onward journey | Unmet care needs | The journey from exclusion and marginalisation to acceptance and belonging |
| Dornelles et al. (2016)^36^ | 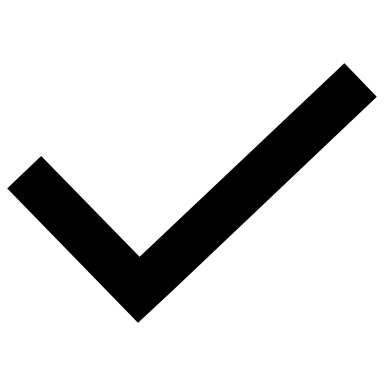 | 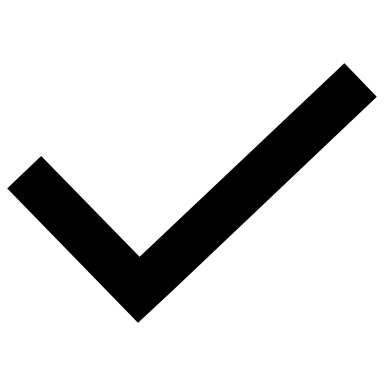 | 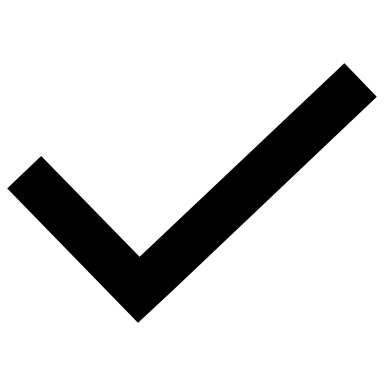 | 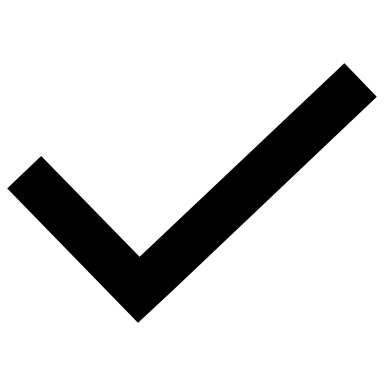 |  |  |  |
| Sonego et al. (2017)^14^ | 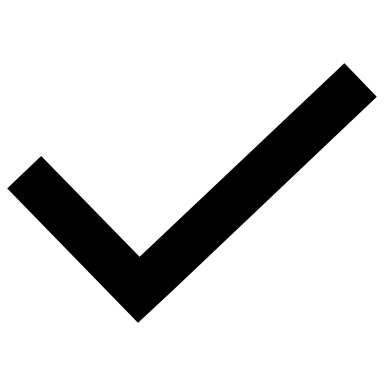 | 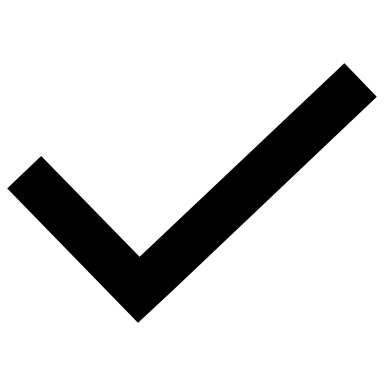 | 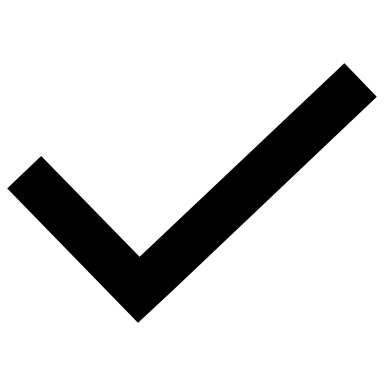 |  |  |  |  |
| Walker et al. (2017)^10^ | 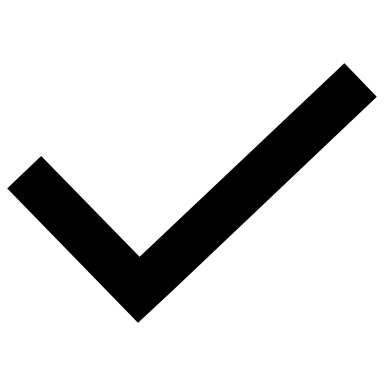 | 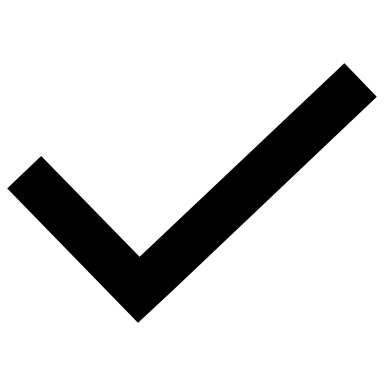 | 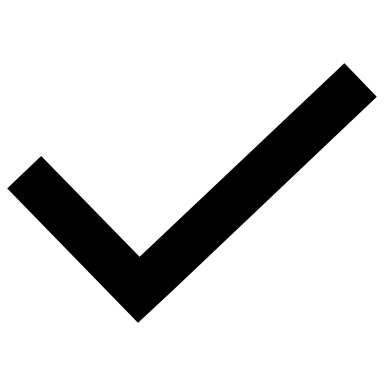 |  |  |  | 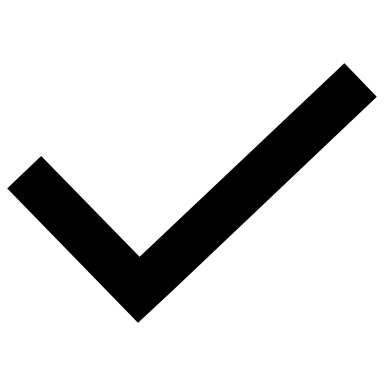 |
| Warmelink et al. (2016)^40^ |  |  |  |  |  | 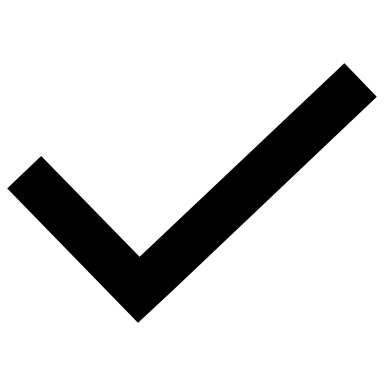 |  |
| French et al. (2015)^12^ | 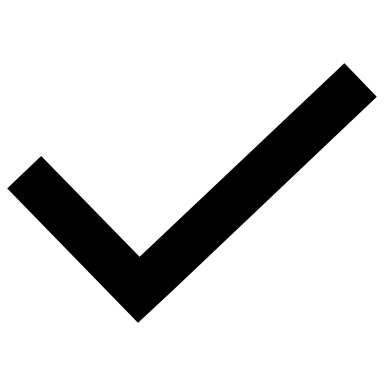 | 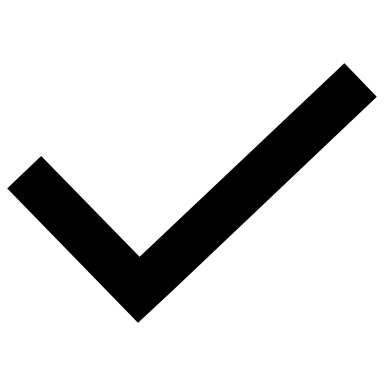 | 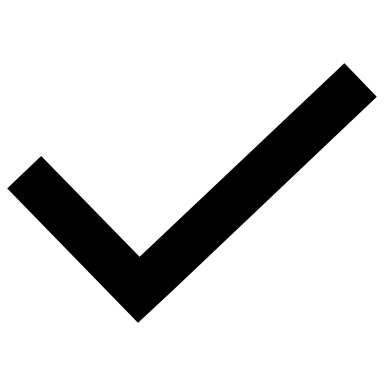 |  |  | 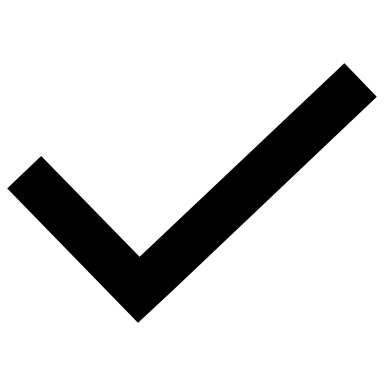 | 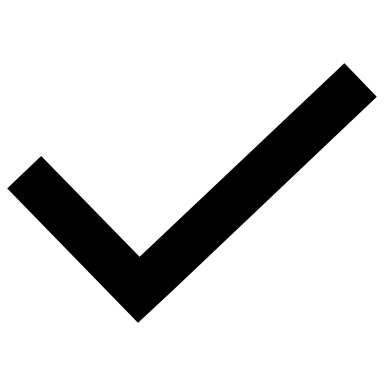 |
| Ranjbar et al. (2015)^9^ | 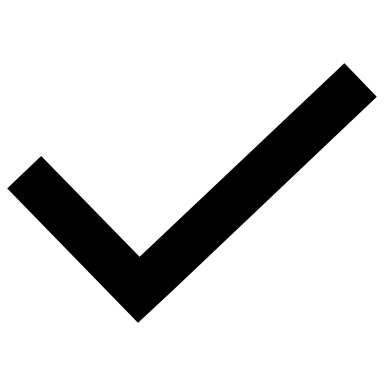 | 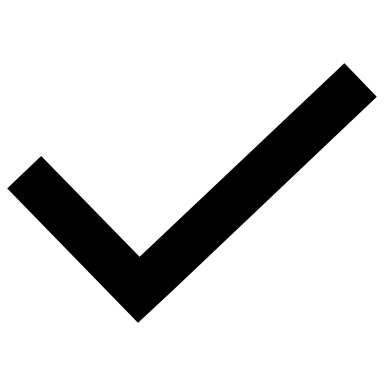 | 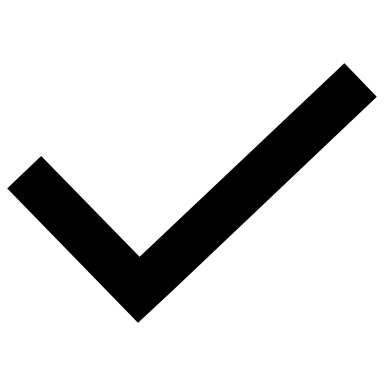 |  |  |  | 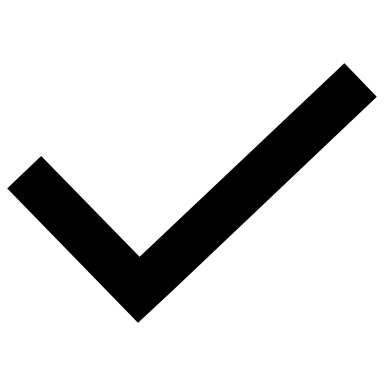 |
| Dornelles et al. (2014)^33^ | 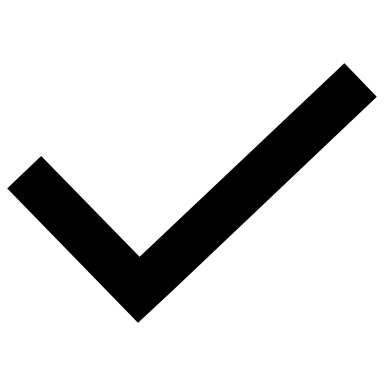 |  | 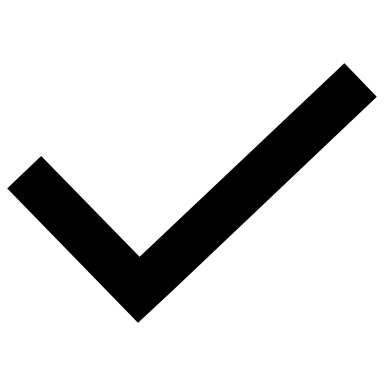 | 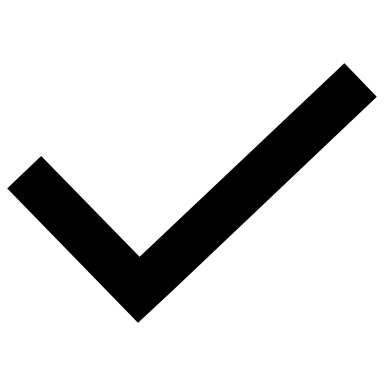 |  |  |  |
| Lin et al. (2013)^34^ | 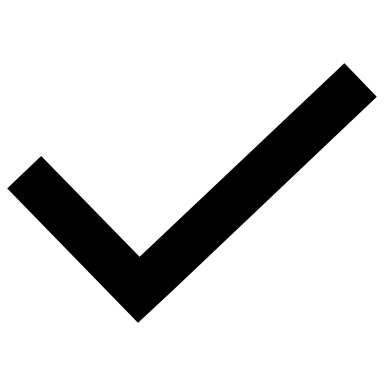 | 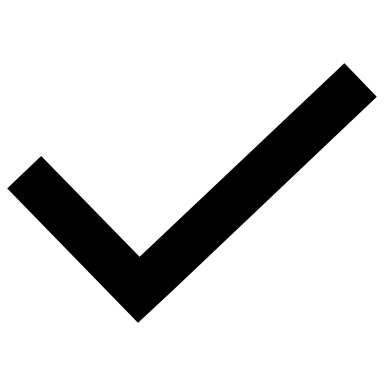 | 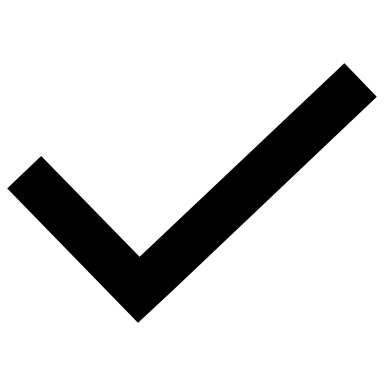 |  |  |  | 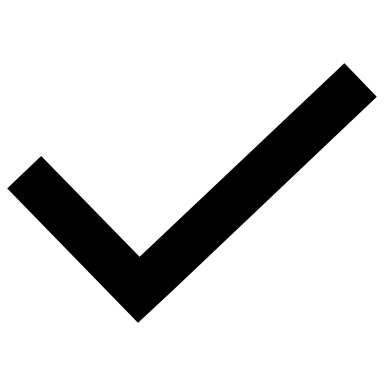 |
| Smorti and Smorti (2013)^29^ | 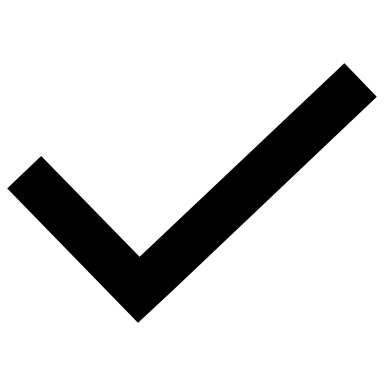 | 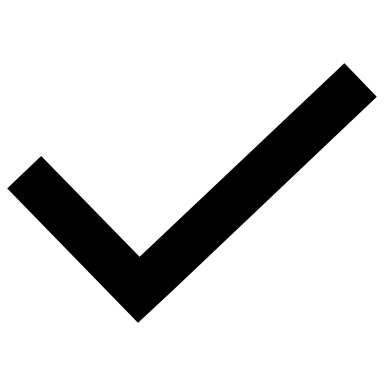 |  |  |  |  | 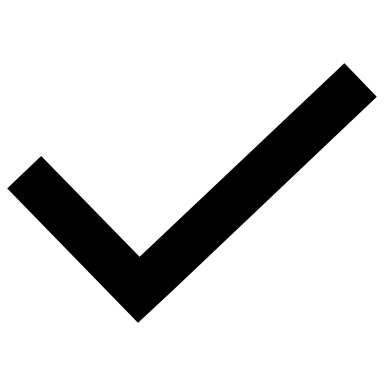 |
| Dornelles and Lopes (2011)^30^ | 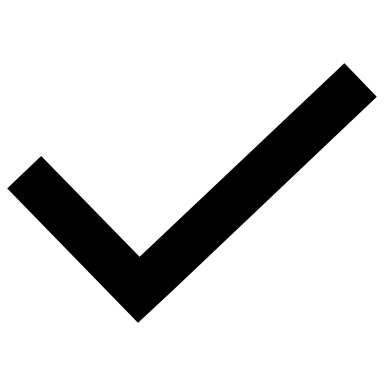 | 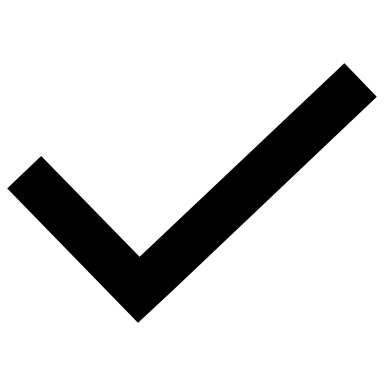 |  |  |  |  | 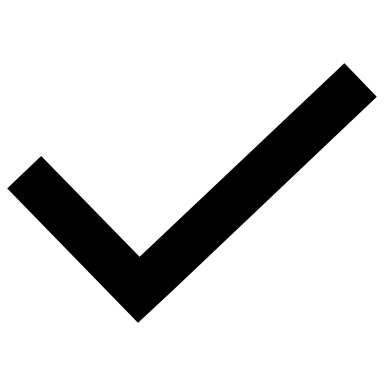 |
| Silva and Lopes (2011)^37^ |  | 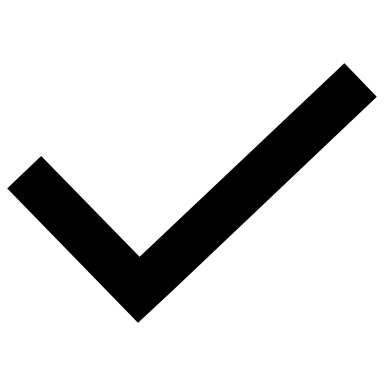 |  |  |  |  | 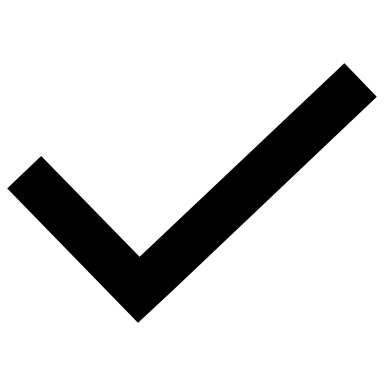 |
| Hayashi and Sayama (2009)^11^ | 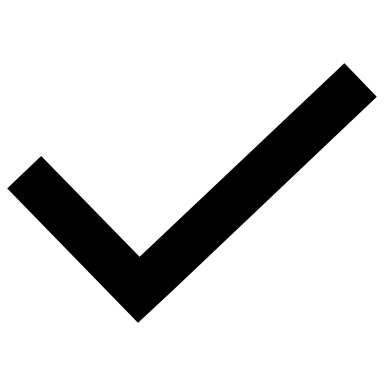 | 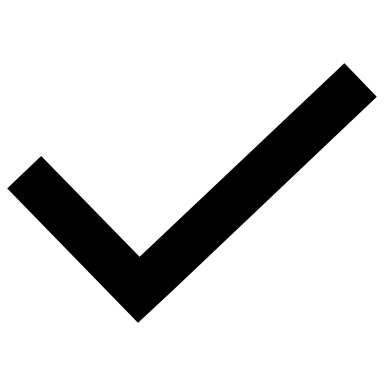 | 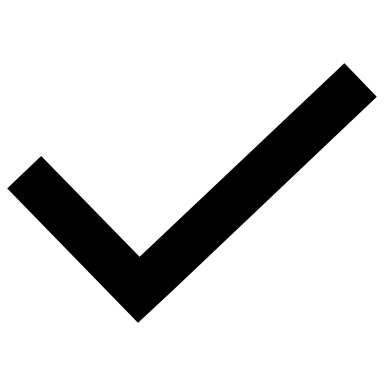 |  |  |  | 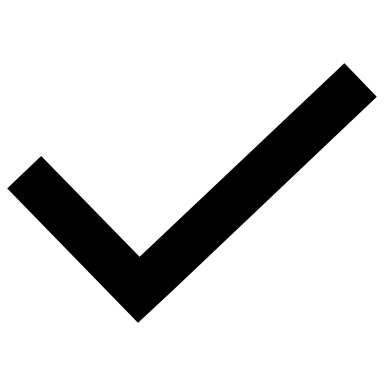 |
